# Downregulation of CYB5D2 is associated with breast cancer progression

**DOI:** 10.1038/s41598-019-43006-y

**Published:** 2019-04-29

**Authors:** Diane Ojo, David Rodriguez, Fengxiang Wei, Anita Bane, Damu Tang

**Affiliations:** 1 Department of Medicine, McMaster University, Hamilton, Canada; 2 Research Institute of St. Joe’s Hamilton, Hamilton, Canada; 3 The Hamilton Center for Kidney Research, St. Joseph’s Hospital, Hamilton, Ontario Canada; 4 The Genetics Laboratory, Institute of Women and Children’s Health, Longgang District, Shenzhen, Guangdong, P.R. China; 5 Department of Pathology and Molecular Medicine, Juravinski Hospital and Cancer Centre, McMaster University, Hamilton, ON Canada

**Keywords:** Breast cancer, Breast cancer

## Abstract

We report here that CYB5D2 is associated with tumor suppression function in breast cancer (BC). CYB5D2 expression was significantly reduced in tamoxifen resistant MCF7 cells and in MCF7 cell-derived xenografts treated with TAM. CYB5D2 overexpression induced apoptosis in MCF7 cells; CYB5D2 knockdown enhanced MCF7 cell proliferation. Using the TCGA and Curtis datasets within the Oncomine database, CYB5D2 mRNA expression was downregulated in primary BCs vs breast tissues and HER2-positive or triple negative BCs vs estrogen receptor (ER)-positive BCs. Using the TCGA and Metabric datasets (n = 817 and n = 2509) within cBioPortal, 660 and 4891 differentially expressed genes (DEGs) in relation to CYB5D2 were identified. These DEGs were enriched in pathways governing cell cycle progression, progesterone-derived oocyte maturation, oocyte-meiosis, estrogen-mediated S-phase entry, and DNA metabolism. CYB5D2 downregulation decreased overall survival (OS, p = 0.0408). A CYB5D2-derived 21-gene signature was constructed and robustly correlated with OS shortening (p = 5.72e-12), and independently predicted BC deaths (HR = 1.28; 95% CI 1.08–1.52; p = 0.004) once adjusting for known clinical factors. CYB5D2 reductions displayed relationship with mutations in PIK3CA, GATA3, MAP3K1, CDH1, TP53 and RB1. Impressively, 85% (560/659) of TP53 mutations occurred in the 21-gene signature-positive BC. Collectively, we provide the first evidence that CYB5D2 is a candidate tumor suppressor of BC.

## Introduction

Breast cancer (BC) is a major cause of cancer related deaths in women. Annually, there are approximately 1.7 million new cases and more than 500,000 fatalities^1^. BC is highly heterogeneous and classified as estrogen receptor-positive (ER+), HER2+, and triple negative (TN; lack of ER, progesterone receptor (PR), and HER2). Advances in profiling of gene expression resulted in grouping of BC into five subtypes - luminal A and B (ER+), basal-like, HER2-enriched, and claudin-low^2–5^. Combination of copy number alteration and gene expression has led to further divide breast cancer into 10 sub-groups^6^. BC is associated with a set of frequent mutations: PIK3CA, TP53, GATA3, MAP3K1, ESR1, as well as FOXA1; these mutations play critical roles in BC progression^7–10^. The above knowledge not only advances our understanding of BC, but also results in better patient management through stratification of patients for specific treatment options. Despite this improvement, BC remains a top health issue for women, illustrating the need to search for additional factors contributing to BC.

CYB5D2 (cytochrome b5 domain containing 2) was reported as a new tumor suppressor in cervical cancer^11^. The protein is also known as neuferricin named after its role in promoting neuron differentiation through inhibiting cell proliferation^12^. As a protein of the membrane-associated progesterone receptors (MAPRs)^13,14^, CYB5D2 contains typical features, including its cytochrome b5 (cyt-b5) domain^11,15,16^. In addition to neuferricin/CYB5D2, the MAPR family includes PGRMC2 (progesterone receptor membrane component 2), PGRMC1, and neudesin^17,18^. PGRMC1 enhances tumorigenesis in several tumor types: breast, ovary, colon, and lung cancers^19–23^, whereas PGRMC2 involvement in tumorigenesis remains relatively unclear^24,25^. Upregulation of PGRMC2 was observed in breast cancer^26^. However, PGRMC2 was able to inhibit ovarian cancer (SKOV-3) cell migration *in vitro*^27^, and its downregulation was observed in metastatic endocervical adenocarcinomas of uterus^28^, suggesting the protein as a tumor suppressor. Similarly, CYB5D2 expression was significantly downregulated in cervical cancer; its enforced expression reduced the *in vitro* invasion and *in vivo* lung metastasis of HeLa cells^11^. The *CYB5D2* gene resides at 17p13.2; 17p13.2–13.3 is lost in 50% of breast cancer^29^, indicating that CYB5D2 may be a novel tumor suppressor of BC.

In support of this possibility, we report here a significant reduction of CYB5D2 expression following the progression of tamoxifen resistance both *in vitro* and *in vivo* as well as in more than 3000 primary BCs. CYB5D2 downregulation is correlated with mutations in PIK3CA, GATA3, MAP3K1 and TP53 as well as reductions in overall survival (OS) of breast cancer.

## Methods

### Tissue culture and the development of tamoxifen-resistant cells

MCF7 cells were purchased from the American Type Culture Collection (ATCC)^30^. Cell were cultured in DMEM accompanied with 10% fetal bovine serum and 1% Penicillin-Streptomycin (Life Technologies, Burlington, ON). Tamoxifen resistant MCF7 (TAM-R) cells were developed by continuous culture of MCF7 cells in phenol-red-free DMEM media in the presence of 1 μM of 4-hydroxyl-tamoxifen (Sigma Aldrich, Oakville, ON) for 12 months^30^. The TAM resistance status was confirmed.

### TUNEL apoptotic detection

MCF7 cells were seeded in chamber slide and transfected transiently with either GFP or GFP-CYB5D2 for 48 hours. TUNEL procedures were then carried out with a TUNEL kit (Abcam) following the manufacturer’s instructions.

### Knockdown of CYB5D2 and proliferation assay

MCF7 cells were transfected using lentivirus-based (control: CTRL) shCTRL or shCYB5D2 (a pool of three individual knockdown constructs; Santa Cruz); the knockdown was confirmed. MCF7 shCTRL and MCF7 shCYB5D2 cells (5 × 10^4^ cells/well) were seeded in a 6-well tissue culture plate with cell numbers counted every three days for nine days.

### Western blot

Cells lysates were produced in a lysis buffer consisting of 20 mM Tris (pH 7.4), 150 mM NaCl, 1% Triton X-100, 1 mM EGTA, 1 mM EDTA, 25 mM sodium pyrophosphate, 0.1 mM sodium orthovanadate, 1 mM β-glycerophosphate, 1 mM NaF, 2 μg/ml leupeptin, 1 mM PMSF and 10 μg/ml aprotinin (Sigma Aldrich, Oakville, ON). Total cell lysate protein (50 μg) was separated on an SDS-PAGE gel and transferred onto nitrocellulose membranes (Amersham, Baie d’Urfe, QC), which were blocked with skim milk (5%) followed by incubation with antibodies to CYB5D2 (1:1000) or actin (Santa Cruz, 1:1000) at 4 °C overnight. Signal was developed using HRP-conjugated secondary antibodies and an ECL Western Blotting Kit (Amersham, Baie d’Urfe, QC)^30^. We quantified protein bands with ImageJ (National Institutes of Health).

### Determination of TAM-derived cytotoxicity

Cells (10^5^ cells/well) were first seeded into a 6 well plate with phenol-red free DMEM media, and cultured for 2 days prior to treatment with either 3 μM TAM or DMSO (1:1000) in serum-free media for 48 hours. Cells were then cultured in compete medium without TAM for 96 hours^31^, followed by fixation in a solution containing 2% formaldehyde and 0.2% glutaraldehyde for 20 minutes prior to addition of a crystal violet solution (0.5% Crystal violet, 20% methanol, 150 mM NaCl) for 30 minutes. The plates were then washed in water and allowed to dry, after which images were obtained. The staining was then released using 2 mL of 33% acetic acid for quantification by measuring absorbance at 550 nm^30^.

### Treatment of xenograft tumor with TAM

Four to five week old ovariectomized nude mice had an 0.72 mg estrogen pelle inserted. 3 × 10^6^ MCF7 cells were implanted into the flank of each mouse after which animals were randomly divided into two groups. Half received a 5 mg tamoxifen pellet and the other half served as controls. Animals were maintained for 28 weeks or until endpoint was reached (1000 mm3). Tumor volumes were determined using a caliper according to the standard formula: L × W^2^ × 0.52, where L and W are the longest and shortest diameters, respectively. All animal work was performed according to protocols approved by the McMaster University Animal Research Ethics Board^30^.

### Immunohistochemistry (IHC) analysis of CYB5D2 expression

Slides were prepared from xenograft tumors treated with and without TAM. IHC staining was performed according to our published protocol^32^. Briefly, slides were deparaffinized in xylene, cleared in ethanol series, and heat-treated for 20 minutes in a food steamer with sodium citrate buffer (pH = 6.0). Antibody specific for CYB5D2 (1:600) was incubated with the sections overnight at 4 °C. Biotinylated secondary IgG and Vector ABC reagent (Vector Laboratories, Burlington, ON) were added subsequently according to the manufacturer’s protocol. Chromogen reaction was performed with diaminobenzidine, and counterstained with hematoxylin^30^. Slides were scanned using a ScanScope and analyzed using ImageScope software (Aperio, Vista, CA). Scores were obtained using the ImageScope software and converted to HScore using the formula [(HScore = % positive X (intensity + 1)]^11,32,33^.

### ER promoter assay

Cells were transfected with the ERE-luciferase reporter (Addgene, Cambridge, MA), pCH110-lacZ plasmid along with CYB5D2 or GFP (green fluorescent protein) vector using Lipofectamine 2000 (Life Technologies, Burlington, ON). After 48 hours cell lysates were assayed for luciferase and β-galactosidase activities. Luciferase activity was normalized to β-galactosidase activity^30,31^.

### Real time PCR analysis

RNA isolation and reverse transcription were carried out using TRIZOL and superscript III reagents (Life Technologies, Burlington, ON) according to the manufacturer’s instructions. Briefly, 2 µg of RNA was converted to cDNA at 65 °C for 6 minutes followed by a 2-minute incubation on ice, 25 °C for 11 minutes, 50 °C for 60 minutes and 70 °C for 15 minutes. Real time PCR primers used include CYB5D2 (F: 5′-GACCGGGGACTGTTCTGAAG-3′; R: 5′-TAGAACCGTCCTGTCACCCT-3′) and Actin (F: 5′-ACCGAGCGCGGCTACAG-3′; R: 5′-CTTAATGTCACGCACGATTTCC-3′). Reactions were performed using the ABI 7500 Fast Real-Time PCR System (Applied Biosystems, Burlington, ON) in the presence of SYBR-green. All samples were run in triplicate^30^.

### Measurement of CYB5D2 mRNA levels

The mRNA expression data of CYB5D2 were extracted from TCGA^34^, Curtis^6^, Finak^35^, and Karnoub^36^ within Oncomine (Compendia Bioscience, Ann Arbor, MI). The genomic mutational data of TP53, PIK3CA, GATA3, MAP3K1, and other genes were retrieved from the Curtis and TCGA-Cell datasets within cBioPortal (http://www.cbioportal.org/)^37,38^. A variety of statistical methods were used to examine CYB5D2 expression and its association with OS (see the section of Statistical analysis). Data related to CYB5D2-associated co-alteration of mutations and gene expression were extracted from the Metabric (n = 2509) and TCGA-Cell (n = 817) datasets within the cBioPortal database^39^. A signature consisting of CYB5D2 reduction and a set of genomic mutations was derived using the Cox regression model. Furthermore, a signature consisting of 21 genes was established using differentially expressed genes (DEGs) related to CYB5D2 downregulation; this was achieved by inputting these DEGs into the Cox model to select for their contributions to hazard ratio (HR) by either forward addition or backward elimination using SPSS Statistics version 23^40^.

### Pathway enrichment analysis

The R packages of Reactome and GAGE as well as Ingenuity Pathway Analysis (IPA) were selected to analyze pathways that were enriched in the differentially expressed genes (DEGs) with the gene ontology (GO) and Kyoto Encyclopedia of Genes and Genomes (KEGG) databases.

### Statistical analysis

Statistical analysis was performed using Student’s t-test. Kaplan-Meier surviving curves, log-rank test, receiver-operating characteristic (ROC) curve, univariate and multivariate Cox proportional hazards regression analyses^40^ were performed using the R survival package and SPSS Statistics version 23. p < 0.05 is considered statistically significant^39^.

## Results

### Downregulation of CYB5D2 during BC tumorigenesis

**Figure 1:**
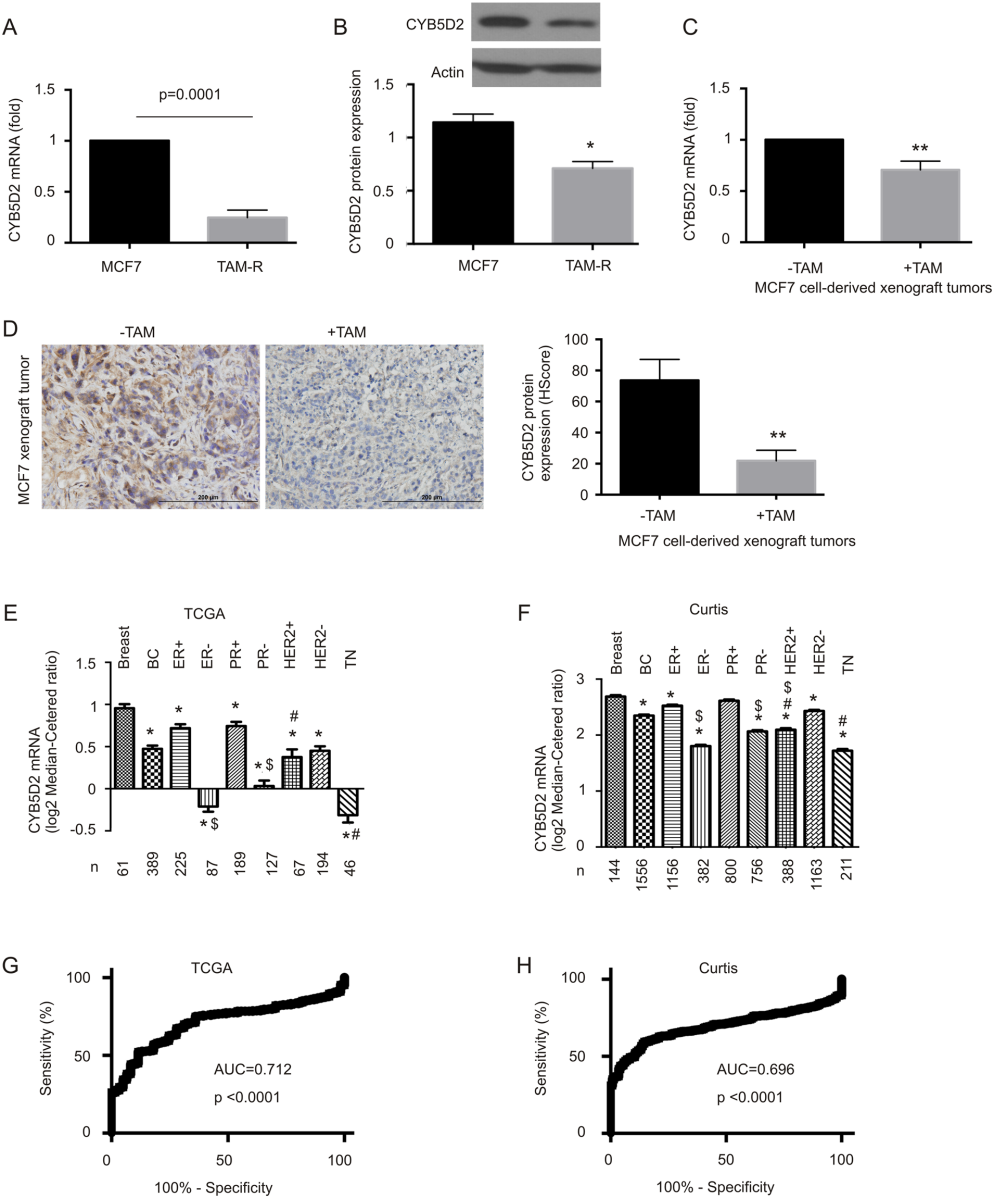
Downregulation of CYB5D2 associates with BC tumorigenesis. (**A**) Real time PCR amplification of CYB5D2 mRNA in MCF7 and TAM-R cells. β-actin was used as an internal control. CYB5D2 mRNA levels were normalized to those of β-actin. Experiments were repeated three times; means ± SD (standard deviation) were graphed. Statistical analysis was performed using Student’s t-test (2-tails). (**B**) Western blot analysis of CYB5D2 protein expression in MCF7 and TAM-R cells. Experiments were repeated three times; typical images are provided (please see Fig S14 for the non-cropped Western blot). CYB5D2 protein expression was normalized to Actin; means ± SD are graphed; *p < 0.05 determined by 2-tailed Student’s t-est. (**C**,**D**) MCF7 cell-derived xenograft tumors were generated in NOD/SCID mice, followed by treatment with and without TAM. CYB5D2 expression in treated (+TAM) and untreated (−TAM) xenograft tumors (n = 5 for each group) was determined by real time PCR (**C**) and IHC (**D**). Typical IHC images are presented. **p < 0.01 in comparison to untreated tumors (2-tailed Student’s t-test). (**E**,**F**) CYB5D2 mRNA expression data were extracted from the datasets of TCGA (**E**)^34^ and Curtis (**F**)^6^. Mean ± SD for the indicated BC subtypes are graphed. *p < 0.05 by an unpaired, two-tailed, welch-corrected t-test; *p < 0.05 in comparison to normal breast tissues (Breast); ^$^p < 0.05 in comparison to the respective ER+ and PR+ tumors; and ^#^p < 0.05 in comparison to ER+ breast tumors. (**G**,**H**) Receiver-operating characteristic (ROC) curves of normal breast tissues versus primary breast tumors were derived from the indicated datasets. AUC: area under the curve.

Our recent identification of CYB5D2 as a tumor suppressor in cervical cancer^11^ as well as its genomic location at 17p13.2; this region is frequently lost in breast cancer^29^; led us to study the possible involvement of CYB5D2 in BC. We have recently derived a Tamoxifen-resistant (TAM-R) line from MCF7 cells (Fig. S1), and detected reductions of CYB5D2 mRNA and protein expression in TAM-R cells (Fig. 1A,B). The downregulation was also demonstrated in MCF7 cell-derived xenograft tumors treated with TAM compared to xenografts produced in untreated mice by either real-time PCR (Fig. 1C) or IHC (Fig. 1D). Addition of TAM decreased MCF7 empty vector (EV) cell-derived xenograft tumor growth^31^. By taking advantage of large datasets of BC gene expression available from the Oncomine database, we showed a significant decrease in CYB5D2 mRNA in BC compared to normal breast tissues in two large patient cohorts (Fig. 1E,F) and two small BC populations (Fig. S2). A further decrease in CYB5D2 expression was demonstrated in aggressive versus less aggressive BC sub-types: ER- versus ER+, PR- versus PR+, and HER2+or TN versus ER+in 2 large BC cohorts, the TCGA^34^ and Curtis datasets^6^ (Fig. 1E,F). In both cohorts, CYB5D2 downregulations separate BC from breast tissues with area under curve (AUC) 0.712 and 0.696, respectively (Fig. 1G,H). Furthermore, CYB5D2 downregulation is associated with a rapid course of overall survival (OS) reduction in BC, including either ER+ or PR+BC (Fig. S3A–C).

We subsequently characterized CYB5D2 downregulation-associated decreases in OS with the Curtis data (n = 1980) (cBioPortal)^6,41^. The dataset was retrieved from cBioPortal; tumors/patients were subsequently separated into a group without CYB5D2 reduction and a group with CYB5D2 downregulation at the levels below 1 standard deviation (SD, −1SD), −1.5 SD, or −2SD from the reference population mean which was derived from tumors that are diploid for the intended gene or the tumor population (http://www.cbioportal.org/faq.jsp). In comparison to BCs without the CYB5D2 downregulations, those with the downregulations are associated with significant reductions in OS within the initial follow-up period of 120 months (Fig. S4).

**Figure 2:**
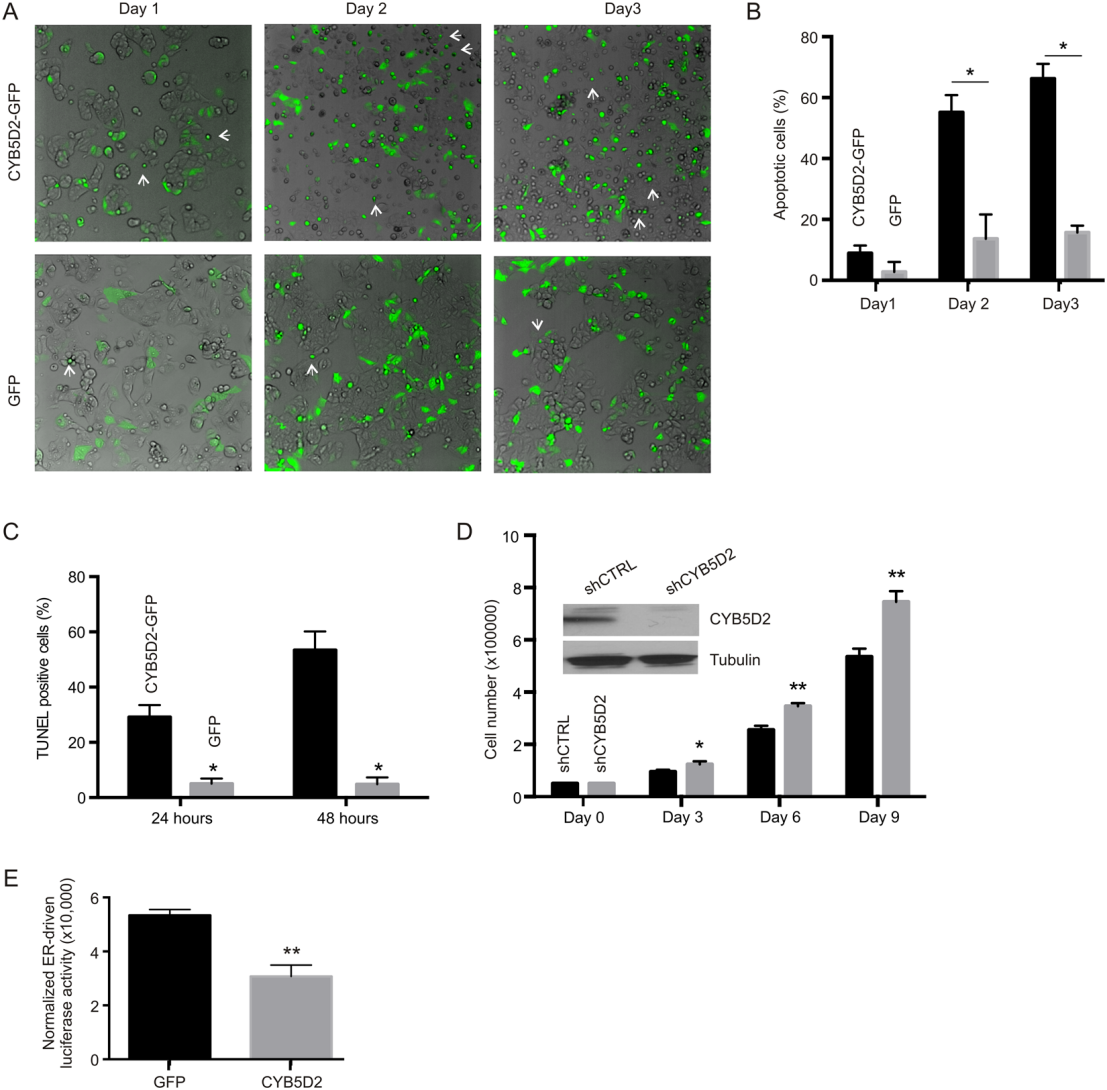
CYB5D2 inhibits MCF7 cell proliferation. (**A**) MCF7 cells were transiently transfected with either CYB5D2-GFP or GFP. Cells were imaged daily at 5 randomly selected fields. The transfection efficiency of CYB5D2-GFP was comparable to that of GFP. Experiments were repeated three times; typical images for one repeat are shown. (**B**) All GFP-positive cells and possible apoptotic cells (see white arrows for typical apoptotic cells) in 5 randomly selected fields were counted; no less than 300 GFP-positive cells for each transfection per time point were counted. Rounded cells (potential apoptotic cells) were then calculated and graphed; *p < 0.05 (2-tailed Student’s t-test) in comparison to the respective GFP transfection. (**C**) MCF cells were transiently transfected with GFP or CYB5D2-GFP for 24 and 48 hours. TUNEL staining was then performed. Cells positive for both TUNEL (red) and GFP as well as positive for GFP only were counted from 5 randomly selected fields; a total of 300–400 cells for each cell type were counted. TUNEL-positive cells are expressed as % of GFP-positive cells. Experiments were repeated three times. Means ± SD are graphed. *p < 0.05 (2-tailed Student’s t-test) in comparison to the respective GFP transfection. (**D**) Stable knockdown of CYB5D2 in MCF7 cells using either shCTRL (control) or shCYB5D2 lentivirus (inset). MCF7 shCTRL and MCF7 shCYB5D2 cells were seeded in 6-well tissue culture plates (5 × 10^4^ cells/well); cell numbers were counted every three days. Experiments were repeated three times. Means ± SD are graphed. *p < 0.05 (2-tailed Student’s t-test) in comparison to MCF7 shCTRL cells. (**E**) 293T cells in 24-well tissue culture plates were transiently transfected in triplicates with a cocktail containing an ER enhancer reconstituted promoter luciferase plasmid plus a β-Gal (galactosidase) vector together with either GFP or CYB5D2 expression plasmid. Luciferase activity was normalized to that of β-Gal. Experiments were repeated three times; means ± SD are graphed; **p < 0.01 in comparison to GFP by 2-tailed Student’s t-test (2-tails).

To investigate the impact of CYB5D2 in BC tumorigenesis, we have made a major effort to stably express CYB5D2 in MCF7 cells. This approach seemed feasible, as CYB5D2 was stably expressed in HeLa cells^11^. However, despite multiple tries by two individuals, a MCF7 cell line stably expressing CYB5D2 could not be established, suggesting that CYB5D2 potently inhibits MCF7 cell viability or proliferation. To study this possibility, we transiently expressed a CYB5D2-GFP (green fluorescence protein) fusion protein or GFP in MCF7 cells, and noticed a large number of rounded-up CYB5D2-GFP cells compared to GFP cells (Fig. 2A,B), indicative of possible apoptosis in MCF7 cells expressing CYB5D2-GFP. Indeed, we detected 29.2% and 53.4% of the CYB5D2-GFP cells as TUNEL-positive 24 hours and 48 hours following transient transfection, respectively (Figs 2C, S5). To examine the effects of CYB5D2 in MCF7 cell growth, CYB5D2 was knocked-down in MCF7 cells (Fig. 2D, inset); knockdown of CYB5D2 significantly enhanced MCF7 cell proliferation (Fig. 2D). The ER + MCF7 cells require ER signaling to survival. Of note, CYB5D2 significantly reduced ER-derived transcription activity based on the luciferase activity driven by an ER enhancer reconstituted SV40 promoter (Fig. 2E). Taken together, we provide the first *in vitro*, *in vivo*, as well as clinical evidence suggesting functional downregulations of CYB5D2 during BC tumorigenesis.

### CYB5D2 downregulation associates with mutations in the major BC contributing genes

It becomes clear that combination of alterations in gene expression with those in genome more precisely revealed critical events of tumor evolution^42^. For incidence, gene expression plus copy number changes resulted in detailed clarification of BC into 10 sub-groups^6^. We thus analyzed CYB5D2 downregulation-associated genomic alterations. With CYB5D2 reduction at the above three levels (Fig. S4), there were no significant co-alterations in copy number variations defined at q-value (false discovery rate) < 0.05 in the Metabric dataset (n = 2509, cBioPortal). On the other hand, we extracted a set of genes with co-alterations in mutation at q < 0.05 (Table S1). It is interesting that those co-mutated genes relative to CYB5D2 downregulation at −1.5 SD or −2SD include the most commonly mutated genes in BC, TP53, CDH1, GATA3, PIK3CA, and MAP3K1 (Table S1)^6,8,34,41,43^. Co-alterations in mutations for either RB1 or BRCA1 also occurred with CYB5D2 reduction at the −1SD level (Table S1).

**Figure 3:**
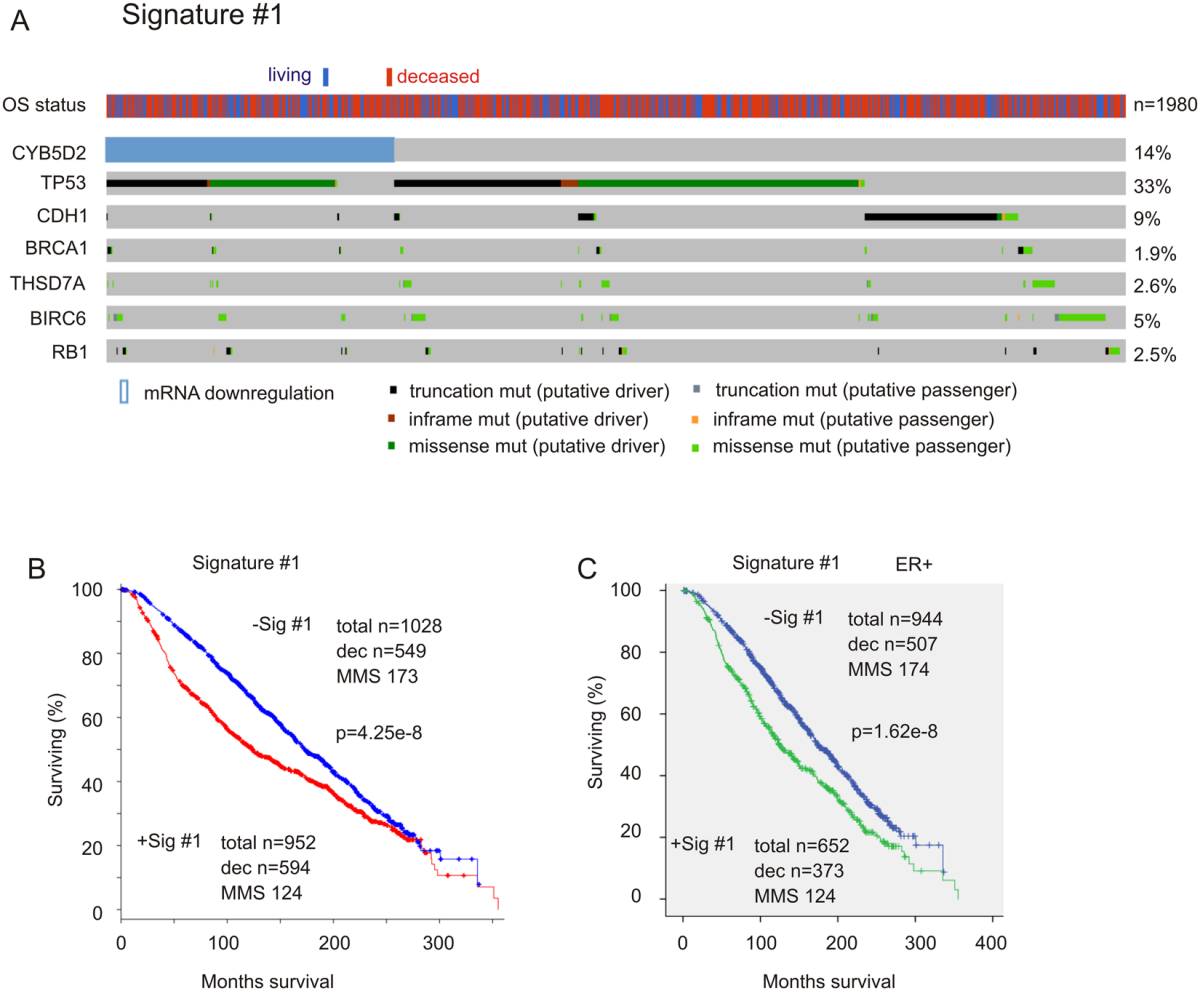
CYB5D2-derived signature #1 associates with reductions in overall survival (OS) in BC patients. (**A**) Data were retrieved from the Curtis dataset (n = 1980)^6^. CYB5D2 mRNA reduction at the −1.5 SD level along with the mutations in the indicated genes are shown. Individual columns are for individual patients. The OS status is also included. Patients with the indicated alterations are shown. (**B**,**C**) The impact of Signature #1 on OS in the entire BC population and ER + subpopulation of the Curtis dataset (n = 1980)^6^. Statistical analysis was performed using Log-rank test. Dec: number of deceased cases; MMS: median months survival.

We then examined whether these genomic alterations will strengthen the effects of CYB5D2 downregulation on OS shortening. Based on the association of CYB5D2 downregulations with an OS decrease (Fig. S4), CYB5D2 reduction at the −1.5 SD level was chosen for further analyses. By selection for contributions to CYB5D2 downregulation-associated decreases in OS, we established a signature (Signature #1) consisting of CYB5D2 reduction and the mutations in TP53, CDH1, BRCA1, THSD7A, BIR6, and RB1 (Table S2; Fig. 3A). Signature #1 significantly correlates with a reduction of OS in BC and ER-positive breast cancer (the Curtis dataset, n = 1980) (Fig. 3B,C). Mutations in TP53 occur most frequently in Signature #1 (Fig. 3A) and contribute to Signature #1’s correlation with OS reductions. Removal of TP53 decreased the signature’s potency, nonetheless, the signature retains association with OS shortening (control cases n = 1145, deaths n = 640, median months survival 169, and 95% CI: 159–181; risk individuals n = 361, deaths n = 241, median months survival 124, and 95% CI: 114–149; p = 4.33e-5). Removal of other individual components also decreased the association (data not shown), supporting their unique contributions to Signature #1. Additionally, Signature #1 independently predicts the risk of BC fatality (HR 1.328, 95% CI 1.131–1.560, p = 5.3e-4) following adjusting for cellularity, age at diagnosis, Neoplasm Histologic Grade, Integrative Cluster, tumor size, Nottingham prognostic index, and tumor stage. The signature remains an independent risk factor for BC deaths after removal of TP53 (HR 1.217, 95% CI 1.041–1.422, p = 0.01379). Additionally, Signature #1 associates with decreases in OS and DFS (disease-free survival) during 80-months follow-up in an independent TCGA-Cell cohort (n = 817)^34^ (Fig. S6). Collectively these findings suggest an important connection between CYB5D2 and other oncogenic factors involved in BC pathogenesis.

### Identification of CYB5D2-related genes

To further characterize CYB5D2’s involvement in BC pathogenesis, we have determined the DEGs (differentially expressed genes) relevant to CYB5D2 downregulation (−1.5 SD) in both the TCGA-Cell (n = 817)^34^ and Metabric (n = 2509) datasets within cBioPortal. DEGs have been selected at q-value < 0.001. In the Metabric and TCGA-Cell datasets, 4981 and 660 DEGs were respectively identified (Tables S3A,B). Of the 660 DEGs extracted from the TCGA-Cell dataset, 471 (71.4%) are found in DEGs retrieved from the Metabric dataset (Table S3C). Furthermore, these 471 genes display the same directionality of alterations (downregulation and upregulation) in both datasets (data not shown).

### Building a 21-gene signature

**Figure 4:**
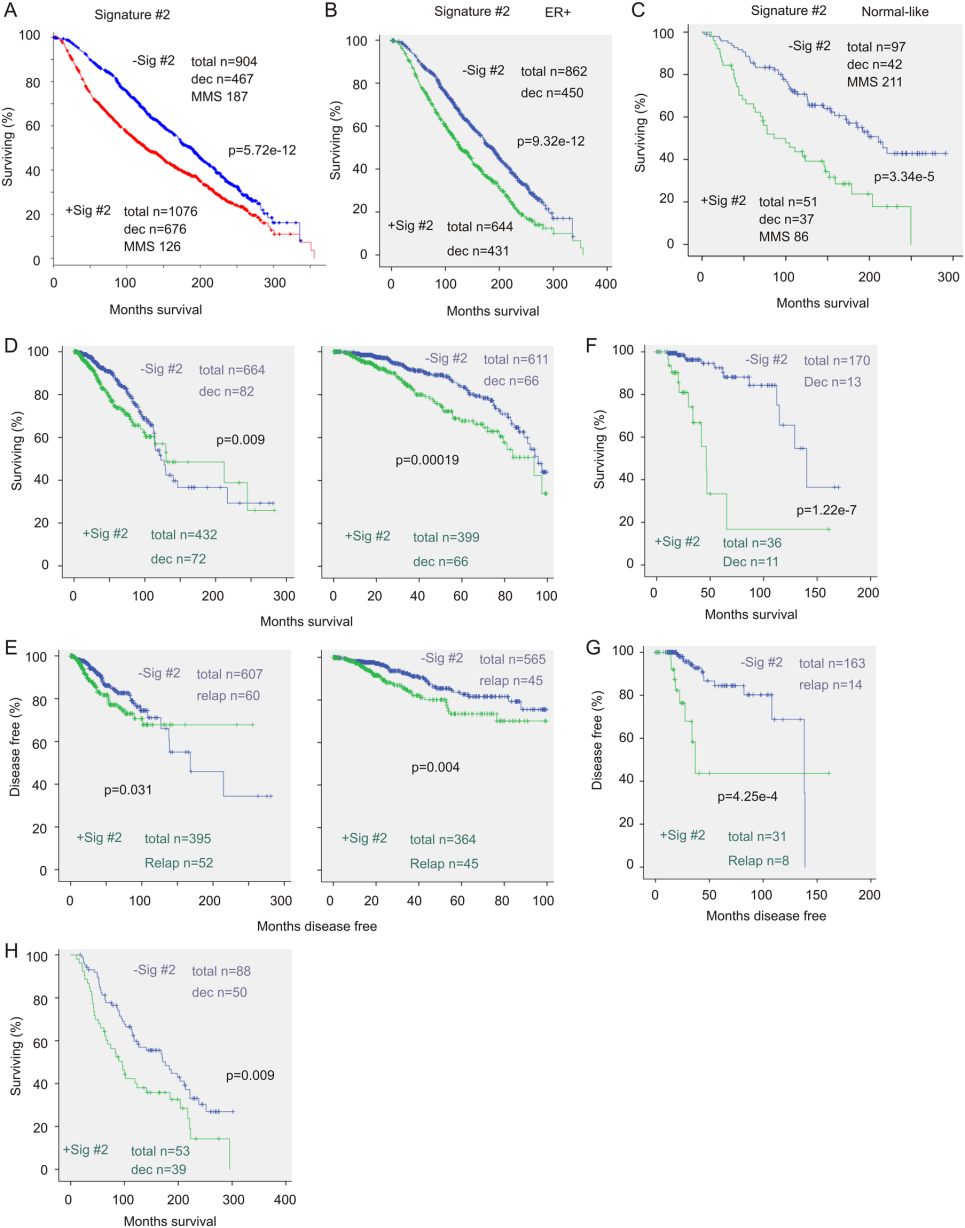
Signature #2 correlates with reductions in OS in patients with breast cancer. The impact of Signature #2 on OS in the entire BC population (**A**), ER + subpopulation (**B**), and normal-like subclass (**C**) within the Curtis dataset (n = 1980)^6^ was determined. (**D–G**) Data from the TCGA-Cell dataset (n = 817)^34^ was used to evaluate the impact of Signature #2 on OS (**D**,**F**) and DFS (**E**,**G**) for the entire population in the indicated follow-up period (**D**,**E**) as well as for lobular breast cancers (**E**,**F**,**H**) Data from the Curtis dataset (n = 1980)^6^ were analyzed for the effects of Signature #2 on OS for patients with lobular breast cancer. Statistical analysis was performed using Logrank Test. dec: deceased cases; MMS: median months survival; relap: relapse cases. For breast tumor composition in both datasets, please see Table S7.

To further analyze those DEGs, we selected DEGs that displayed alterations no less than that of CYB5D2. From the Metabric-DEGs, 98 genes were selected with 64 downregulation and 34 upregulation (Table S3D). We then imputed these DEGs in the Cox model using either addition (forward) or elimination (backward) of covariates to select DEGs based on their contributions to hazard ratio (HR). A 21 gene signature (Signature #2) was the result (Table 1; Fig. S7). Signature #2 robustly correlates with decreases of OS in BCs in the Curtis cohort (n = 1980, p = 5.72e-12) and the ER-positive subpopulation (n = 1560, p = 9.32e-12) (Fig. 4A,B). In the ER-negative subpopulation, Signature #2 is on a border line (p = 0.077) for a significant association with OS reductions (Fig. S8A). We noticed a large proportion of ER-negative tumors (432 of 474) being positive for Signature #2 (Fig. S8A), suggesting that this imbalance in distribution was a cause for the observed non-significant association (Fig. S8A).

**Table 1:** Components of Signature #2^a^.

Gene	Cytoband	Protein	Function	Ref.
APOD^b^	3q29	Apoliproproein D	BC promotion	^51^
NOSTRIN^b^	2q31.1	NO synth traff inducer^d^	suppr pancr cancer progr^e^	^52^
SCUBE2^b^	11p15.3	Signal peptide-CUB-EGF domain-containing protein 2	Suppression of BC	^53^
SLC40A1^b^	2q32	solute carrier family 40 member 1	Association with favorable prognosis in BC patients	^53^
SLC7A2^b^	8p22	solute carrier family 7 member 2	likely involved in BC	^54^
AFF3^b^	2q11.2-q12	AF4/FMR2 family member 3	likely involved in BC	^55^
CYB5D2^b^	17p13.2	CYB5D2/Neuferricin	a tumor suppressor	^11^
FBP1^b^	9q22.3	fructose-1, 6-bisphosphatase 1	Tumor suppressor of BC	^56^
STMND1^b^	6p22.3	stathmin domain containing 1	unknown	
XBP1^b^	22q12.1|22q12	X-box binding protein 1	Enhancing BC tumorigenesis	^57^
C1ORF106^c^	1q32.1	chromosome 1 open reading frame 106	activation of MAPK and NF-κB pathways	^58^
CALML5^c^	10p15.1	calmodulin like 5	promoting BC tumorigenesis	^59^
CBX2^c^	17q25.3	chromobox 2	promoting BC metastasis	^60^
CCNE1^c^	19q12	Cyclin E	Promoting BC progression	^61^
KIF1A^c^	2q37.3	kinesin family member 1A	Association with relapse of ER + BC	^62^
KRT16P3^c^	17p11.2	keratin 16 pseudogene 3	unknown	
LAD1^c^	1q25.1-q32.3	ladinin 1	Association with TN BC	^63^
SLPI^c^	20q12	secretory leukocyte peptidase inhibitor	Promoting angiogenesis in BC	^64^
TTK^c^	6q14.1	Monopolar spindle 1	Promoting mitosis	^65^
UBE2C^c^	20q13.12	ubiquitin conjugating enzyme E2 C	Assoc with poor prognosis in patients with breast cancer	^66^
S100A8^c^	1q21	S100 calcium binding protein A8	Assoc with poor prognosis in patients with BC	^66^

^a^upregulations and downregulations are defined at 1.5 SD away from the population means.

^b^downregulated genes; c: upregulated genes;

^d^Nitric oxide synthase trafficking inducer;

^e^an eNOS interaction partner; suppressing pancreatic cancer progression.

We subsequently examined the impact of Signature #2 on OS in patients with PAM50-classified intrinsic subtypes using the Curtis dataset (cBioPortal). As the luminal subtypes are essentially ER-positive breast tumors^2–5^, we have focused on other intrinsic subtypes: claudin-low, normal-like, basal-like, and HER2-enriched BCs. Among 209 basal-like BCs, 198 were Signature #2-positive; of that, 110 patients died. Signature #2-positive basal-like BCs exhibited comparable OS compared to those of signature-negative (p = 0.659). In HER2-enriched and particularly claudin-low BCs within the follow up period of 160 months, Signature #2-positive BCs display a significantly shorter OS (Fig. S8b,C, right panel). Impressively, Signature #2 significantly correlates with OS decreases in normal-like BCs (Fig. 4C). Similarly, in the largest TCGA provisional cohort (n = 1101), a population containing the TCGA-Cell cohort (n = 817)^34^, Signature #2 associates with decreases in both OS and DFS within the follow-up period of 100 months (Fig. 4D,E). The TCGA cohorts contain an average of 18.8% lobular BC and has been used to profile the genomic and expression landscape of lobular BCs^34^. Of note, Signature #2 correlates with decreases in OS and DFS in lobular BC in TCGA (Fig. 4F,G) and the Curtis cohorts (Fig. 4H). Furthermore, Signature #2 independently predicts OS once adjusting for clinical features (Table 2). Collectively, these observations reveal associations of CYB5D2-derived 21-gene signature with decreases of OS and DFS in HER2-enriched, normal like molecular subtype, and lobular BC.

**Table 2:** Univariate and multivariate cox analysis of CYB5D2-derived Signature #2.

Clin var and sig^a^	Univaraite			Multivariate		
	HR^b^	95% CI^c^	p-value	HR	95% CI	p-value
Signature #2	1.51	1.34–1.7	8.04-e12*	1.28	1.08–1.52	0.004*
Age at diagnosis	1.04	1.03–1.04	2e-16*	1.03	1.03–1.04	2e-16*
Cellularity	1.02	0.94–1.12	0.618	0.99	0.88–1.10	0.828
**Integrat Cluster** ^**d**^						
Cluster 3	0.72	0.55–0.94	0.016*	0.95	0.68–1.34	0.779
Cluster 5	1.56	1.18–2.06	0.002*	1.65	1.17–2.31	0.004*
Neo His G^e^	1.28	1.17–1.41	3.35e-7*	0.75	0.62–0.92	0.005*
N Prog index^f^	1.32	1.26–1.40	2e-16*	1.399	1.23–1.59	1.78e-7*
Tumor size	1.01	1.012–1.018	2e-16*	1.001	1.004–1.013	9.51e-5*
Tumor stage	1.81	1.63–2.02	2e-16*	1.14	0.96–1.35	0.123

^a^Clinical variables and Signature #2.

^b^hazard ratio.

^c^confidence interval.

^d^integrative cluster.

^e^neoplasm histologic grade.

^f^nottingham prognostic index.

### Co-occurrence of Signature #2 with TP53 and RB1 mutations

We observed 80.6% of BCs with CYB5D2 reduction at the −1.5 SD level contained TP53 mutations in the Metabric dataset (Table S1; p = 2.95e-56, q = 5.11e-54); the concordance remains in the Curtis sub-population (p = 1.31e-61, q = 2.26e-59). Interestingly, CYB5D2-derived Signature #2 shows a substantial enrichment in the co-occurrence with 85% (560/659) of TP53 mutations detected in Signature #2-positive BCs (Table 3; p = 3.02e-80, q = 5.22e-78). While RB1 mutations co-occurred only with CYB5D2 reduction at the −1SD level in the Metabric (n = 2509, Table S1) and Curtis cohort (26/49 = 53.1%, p = 1.192e-3, q = 0.0206), 79.6% (39/49) of RB1 mutations were detected in Signature #2-positive tumors in the Curtis dataset (Table 3). Additionally, among 11 co-mutated genes which occurred with Signature #2 (Table 3), 9 were also associated with CYB5D2 reductions (Table S1), suggesting an intimate relationship of these mutations with Signature #2. Indeed, addition of the genomic (mutation) components of Signature #1 did not strengthen Signature #2’s correlation with OS shortening in the Curtis dataset (data not show). Both p53 and pRB proteins are critical inhibitors of cell cycle progression; the observed concordances thus suggest that CYB5D2 plays a role in regulating BC cell proliferation.

**Table 3:** Co-alteration of mutations with Signature #2^a^.

Gene	locus	+Sig#2	−Sig#2	Log R^c^	p-value	q-value
TP53^b^	17q13.1	560 (52.98%)	99 (12.24%)^e^	2.11	3.02e-80	5.22e-78
PIK3CA^b^	3q26.3	362 (34.25%)	433 (53.52%)	−0.64	5.0e-17	4.32e-15
CBFB^b^	16q22.1	21 (1.99%)	71 (8.78%)	−2.14	1.35e-11	7.80e-10
MAP3K1^b^	5q11.2	75 (7.1%)	123 (15.20%)	−1.1	1.53e-8	6.64e-7
GATA3^b^	10p11	94 (8.89%)	136 (16.81%)	−0.92	2.08e-7	7.19e-6
CDH1^b^	16q22.1	70 (6.62%)	102 (12.61%)	−0.93	7.53e-6	2.17e-4
DNAH11	7p21	120 (11.35%)	55 (6.80%)	0.74	4.73e-4	0.0117
RB1^b^	13q14.2	39 (3.69%)	10 (1.24%)	1.58	5.74e-4	0.0124
GLDC	9p22	29 (2.74%)	6 (0.74%)	1.89	9.06e-4	0.0174
SYNE1^b^	6q25	152 (14.38%)	80 (9.89%)	0.54	2.08e-3	0.0359
AKAP9^b^	7q21–22	81 (7.66%)	36 (4.45%)	0.78	2.73e-3	0.0429

^a^CYB5D2 mRNA reduction at levels < −1.5 SD; mutations in co-alteration with Signature #2 were determined using the Curtis dataset (n = 817).

^b^these mutations were co-altered with CYB5D2 downregulation (see Table S1).

^c^log2-based ratio of percentage in altered group/percentage in unchanged group; positive and negative ratios are for co-occurrence and mutual exclusiveness, respectively.

### CYB5D2-associated DEGs affect pathways regulating cell proliferation and DNA metabolism

To gain insight on the potential mechanisms contributing to the tumor suppression functions of CYB5D2 in BC, we analyzed pathways affected by the DEGs relative to CYB5D2 downregulation using the GAGE^44^ and Reactome^45^ packages in R as well as Ingenuity Pathway Analysis. Analysis of the 471 DEGs of both the TCGA-Cell (n = 817) and Metabric cohorts (common-DEGs, Table S3C) with the GAGE package using the KEGG gene sets identified three upregulated gene sets functioning in progesterone-regulated oocyte maturation, cell cycle, and oocyte meiosis (Table 4; Fig. S9; Tables S4A–C). Analysis of the entire 660 DEGs obtained from the TCGA-Cell (TCGA-DEGs) cohort recapitulated the enrichment (Table S4D), indicating all critical DEGs that occurred in TCGA-DEGs were present in Metabric-DEGs. Indeed, the same cell cycle KEGG gene set (hsa04110 cell cycle) was also enriched and upregulated in Metabric-DEGs (Table S4E). Two additional gene sets of DNA replication and ribosome biosynthesis were also enriched and upregulated in the Metabric-DEGs (Table S4E); pathways regulated by both gene sets belong to the core machinery of cell proliferation. In accordance with cell proliferation being critical for tumorigenesis, a cancer-related pathway was upregulated in common-DEGs (Table 4).

**Table 4:** Upregulation of gene sets and pathways in the common-DEGs^a^.

Gene sets^b^	Set size^c^	p-value	q-value
hsa04110 Cell cycle^d^	26	3.01e-5	9.02e-5
hsa04114 Oocyte meiosis^d^	14	0.001583	0.002375
hsa04914 Progesterone-mediated oocyte maturation^d^	11	0.007763	0.007763
hsa05200 Pathways in cancer^e^	16	0.05089	0.05089
GO^f^ :0000278 mitotic cell cycle	62	4.86e-10	1.52e-8
GO:0000279 M phase	57	2.62e-9	4.07e-8
GO:0000087 M phase of mitotic CC^g^	47	4.29e-8	3.33e-7
GO:0000280 nuclear division	47	4.29e-8	3.33e-7
GO:0006259 DNA metabolic process	41	3.62e-4	2.244e-3
GO:0006260 DNA replication	29	7.99e-4	4.127e-3
GO:0006139 NNN^h^ and nucleic acid metabolic process	113	2.998e-3	0.013279
GO:0000075 cell cycle checkpoint	12	5.828e-3	0.022584
GO:0000070 mitotic sist chrom segr^i^	10	0.010869	0.033695
GO:0000819 sist chroma segr^i^	10	0.010869	0.033695
GO:0006281 DNA repair	17	0.01302	0.036693
GO:0006511 ub protein catab process ^j^	19	0.017101	0.044177
GO:0006468 protein AA phosph^k^	29	0.019182	0.045741
IPA pathways and diseases^l^	Overlap^m^	p-value	Mole^q^
Mitotic Roles of Polo-Like Kinase	22.7%(15/66)	7.74e-12	
CC Control of Chromo Replic^n^	28.9%(11/38)	2.63e-10	
Estrogen-mediated S-phase Entry	33.3%(8/24)	2.19e-8	
CC: G2/M DD Checkpoint Reg°	20.4%(10/49)	6.87e-8	
Role of BRCA1 in DDR^p^	15.4%(12/78)	9.28e-8	
Cancer		1.34e-3 to 6.76e-16^r^	414
Organismal Injury and Abnormalities		1.34e-3 to 6.76e-16	420
Reproductive System Disease		1.04e-3 to 6.76e-16	244
Gastrointestinal Disease		1.12e-3 to 2.87e-12	368
Nutritional Disease		3.97e-4 to 6.64e-11	29

^a^Enrichment in gene sets and pathways was performed using the GAGE package in R and Ingenuity pathway analysis (IPA); ^b^the indicated gene sets were upregulated in the common-DEGs; ^c^number of genes in the common DEGs that are enriched in the individual gene sets; ^d^gene sets enriched in KEGG gene sets; ^e^gene sets enriched in the KEGG disease gene sets; ^f^enriched gene sets in the Gene Ontology (GO) database; ^g^cell cycle; ^h^nucleobase, nucleoside, nucleotide; ^i^sister chromatid segregation; ^j^ubiquitin-dependent protein catabolic process; ^k^protein amino acid phosphorylation; ^l^pathways and diseases affected in common-DEGs were determined using IPA; ^m^number of common-DEGs/number of pathway genes x 100 with the respective number of genes indicated in parentheses; ^n^Cell cycle Control of Chromosomal Replication; ^o^Cell Cycle: G2/M DNA Damage Checkpoint Regulation; ^p^DNA damage response; ^q^number of molecules involved in the indicated diseases; ^r^p-value range.

By using the GO gene set and the go.sets.hs datasets^44^, gene sets related to multiple aspects of cell cycle, mitotic phase, DNA metabolism, DNA replication, DNA repair, checkpoint activation and others were also significantly enriched in common-DEGs (Table 4; Table S5A). Similar gene sets of GO terms were enriched in TCGA-DEGs (Table S5B,C) and Metabric-DEGs (Table S5D,E).

Consistent with the above gene-set enrichment analyses, pathway enrichment determination using the Reactome package in R^45^ yielded pathways regulated by the above enriched gene sets in common-DEGs, TCGA-DEGs, and Metabric-DEGs (Fig. S10; Table S6A–C). The enriched pathways derived from common-DEGs are centered on processes related with mitosis (Fig. S11). In addition to mitosis, TCGA-DEGs regulate cell cycle and ATR activation which is required for DNA replication in S-phase^46^ (Fig. S12). Furthermore, the Metabric-DEGs participate in three major pathways: S-phase, G1-S phase, and impressively p53-regulated transcription (Fig. S13). Cyclin D1-Cdk4/6 promoted G1 phase progression is a major oncogenic force for BC^47^. The enrichment of cyclin D1-regulated G1 events (Table S6C) and G1 cell cycle transition (Figs. S9, S13) in Metabric-DEGs supports an important role of CYB5D2 downregulation in BC pathogenesis. Additionally, the above pathway analysis is in accordance with the results generated using Ingenuity Pathway Analysis (IPA); IPA was able to pinpoint the DEGs-associated inhibition of CDKN1A (encoding p21^CIP1^ CDK inhibitor) and activation of HER2 and E2F4 (data not shown). Collectively, the above analyses support the activation of cell cycle machinery in CYB5D2 downregulation-associated DEGs.

## Discussion

Evidence suggesting a role of CYB5D2 in BC suppression includes its reported tumor suppression in cervical cancer^11^ and its chromosomal localization at 17p13.2; loss of which occurs frequently in BC^29^. While homodeletion of CYB5D2 can be detected in both the TCGA (8/1093 = 0.7%) and Metabric (4/2051 = 0.2%) datasets, the frequency is low; nonetheless, in both cohorts homodeletion in TP53 was not detected. However, mutation in TP53 occurs in 33% (659/1980) of the population in the Curtis and 34% (281/816) in the TCGA-Cell population. Although mutation in the CYB5D2 genes was undetectable in both, its expression is significantly reduced *in vitro*, *in vivo* and in primary BCs (Fig. 1); importantly, we have shown that CYB5D2 may regulate MCF7 cell viability or proliferation (Fig. 2). Although different genetic alterations are in play in the inactivation of p53 and CYB5D2, both events occur in a strong concordance; no less than 80% of tumors with CYB5D2 reduction < −1.5 SD contain TP53 mutations (Table S1), and 85% of TP53 mutations are in tumors positive for CYB5D2-derived 21-gene signature (Table 3). This concordance suggests collaboration between p53 inactivation and CYB5D2 downregulation in BC tumorigenesis.

Significant co-occurrence of RB1 with CYB5D2 reduction (Table S1) and particularly Signature #2 in which 79.6% (39/49) of RB1 mutations were detected in Signature #2-positive tumors (Table 3) agrees well with the enrichment of CYB5D2 reduction-associated DEGs in cell cycle progression, mitotic phase events, and cell proliferation. On the other hand, the mutual exclusiveness of CYB5D2 downregulation with other major oncogenic events of PIK3CA, MAP3K1, GATA3, and CDH1 (Table 3) implies that CYB5D2 downregulation is a component of the oncogenic processes involving these oncogenic factors. While the association with above genomic alterations suggest mechanisms contributing to CYB5D2-derived tumor suppression in breast cancer, the potential mechanisms underlying the co-occurrence and mutual exclusion of CYB5D2 expression with the above gene mutations are likely complex. DEGs accompanied with CYB5D2 downregulation affect pathways regulating DNA repair (Table 4). This suggests a scenario that downregulation of CYB5D2 leads to genome instability, which facilitates gene mutations. However, this will not explain the selectivity of CYB5D2 downregulation with mutations in tumor suppressor and oncogenes as aforementioned above. With the current knowledge, we suggest that CYB5D2 downregulation selectively associates with genomic alterations via CYB5D2-derived tumor suppression activity. This possibility is consistent with CYB5D2 inducing apoptosis in MCF7 cells (Fig. 2C).

CYB5D2 reduction is associated with a large number of DEGs in primary BC (Table S3). While the underlying mechanisms affecting the gene expression have not been studied, it is possible that CYB5D2 indirectly alters gene expression though its tumor suppression function. Structural wise, CYB5D2 does not have motifs known to directly modulate gene expression^11,16^. Stable expression of CYB5D2 in HeLa cells did not substantially affect gene expression^16^.

Currently, there are approximate 140 driver genes functioning in 12 signaling pathways involving PI3K, MAPK, cell cycle, and DNA damage regulation^48^. These pathways are enriched in CYB5D2 downregulation-associated DEGs, supporting CYB5D2 as a novel tumor suppressor in BC. Nonetheless, it is important to directly examine this notion and to investigate the underlying mechanisms. Currently, we are studying these avenues. However, our observed correlation of CYB5D2 with BC progression and the results generated *in silico* using more than 3,000 patients with BC from two of the most comprehensive BC datasets provide a strong basis to explore CYB5D2-derived novel tumor supressing activities.

It appears that Signature #1 and #2 exhibit a more robust correlation with OS shortening in the Curtis population (Figs 3, 4A–C) compared to the TCGA-Cell cohort (Fig. 4D–H). A potential factor for these observations might be attributable to the difference in cohort composition (Table S7). While additional research is certainly required to determine the biomarker potential of the 21-gene signature, its predictive value in multiple intrinsic subtypes of BC (Figs 4A–C, S8) is not only appealing but also in accordance with its extensive overlap with TP53 mutations, an event that occurs in all intrinsic subtypes of BC. While evidence supports the unique biomarker value of CYB5D2-associated 21-gene signature, its clinical potential needs to be further tested both retrospectively and prospectively in future.

There are several promising multigene signatures available commercially to assess disease recurrence for newly diagnosed patients with different BC types, including Oncotype DX, MammaPrint, EndoPredict, and Prosigna^49,50^. Our signatures were constructed to predict OS and thus could be used together with these multigene panels to improve decision making and patient management.

## Supplementary information

Supplementary Figures S1-S15 and Tables S1-S2

Table S3

Table S4

Table S5

Table S6

Table S7

## Data Availability

We are committed to have all materials and data available to the research community upon publication.
