# Supplementary material for: Downregulation of CYB5D2 is associated with breast cancer progression
Source: Sci Rep. 2019 Apr 29;9:6624. doi: 10.1038/s41598-019-43006-y (PMC6488675; doi:10.1038/s41598-019-43006-y)
Supplement: Supplementary file 1 — Supplementary Figures S1-S15 and Tables S1-S2 [file 41598_2019_43006_MOESM1_ESM.pdf]

Downregulation of CYB5D2 is associated with breast cancer progression

Diane Ojo,[1,2,3] David Rodriguez,[1,2,3] Fengxiang Wei,[4,*] Anita Bane,[5] and Damu Tang [1,2,3,*]

[1]Department of Medicine, McMaster University; [2]Research Institute of St Joe's Hamilton, [3]the Hamilton Center for Kidney Research, St. Joseph's Hospital, Hamilton, Ontario, Canada. [4]The Genetics Laboratory, Institute of Women and Children's Health, Longgang District, Shenzhen, Guangdong, P.R. China; [5]Department of Pathology and Molecular Medicine, Juravinski Hospital and Cancer Centre, McMaster University, Hamilton, ON, Canada

*Correspondence:
Damu Tang
T3310, St. Joseph's Hospital
50 Charlton Ave East
Hamilton, Ontario, Canada
L8N 4A6
Tel: (905) 522-1155, ext. 35168
Fax: (905) 521-6181
E-mail: damut@mcmaster.ca

Fengxiang Wei
50 Aixin Road
Shenzhen, Guangdong
China 518174
Tel.: + 86 13088883678
Email: haowei727499@163.com

Key words: CYB5D2, tumor suppressor, breast cancer, overall survival, disease free survival

Figure S1

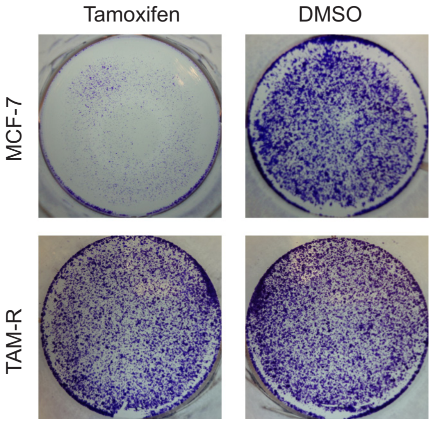

Figure S1. TAM-R cells are resistant to tamoxifen-induced cytotoxicity. MCF7 and TAM-R cells were treated with either DMSO or tamoxifen at 3 M for 2 days, followed by culture in a TAM-free medium for 3 days. Cells were then stained with crystal violet (0.5%). Experiments were repeated at least three times; typical images from one repeat are included.

Figure S2

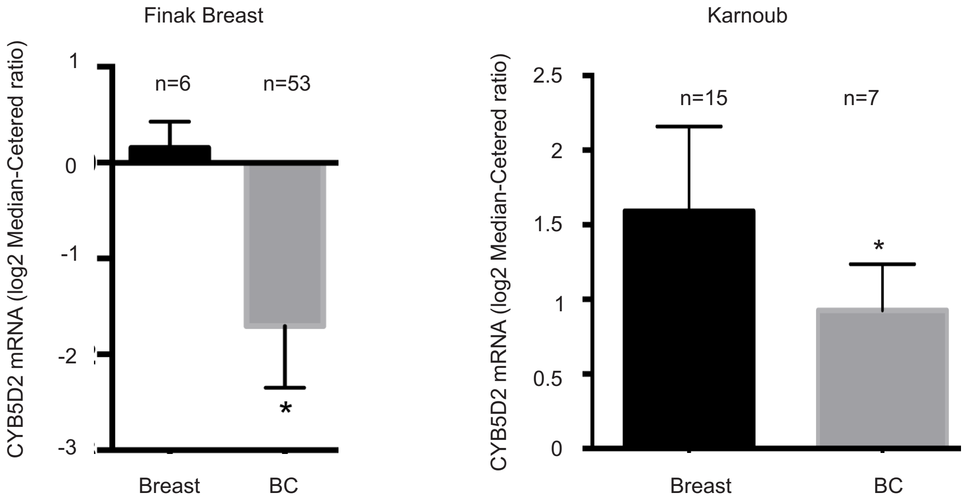

Figure S2. Reduction of CYB5D2 mRNA expression in breast cancer. Data were extracted from the indicated datasets within Oncomine™ (Compendia Bioscience, Ann Arbor, MI). Mean ± SD are graphed. *p<0.05 by a 2-tailed Student's *t*-test in comparison to normal breast tissues (Breast).

Figure S3

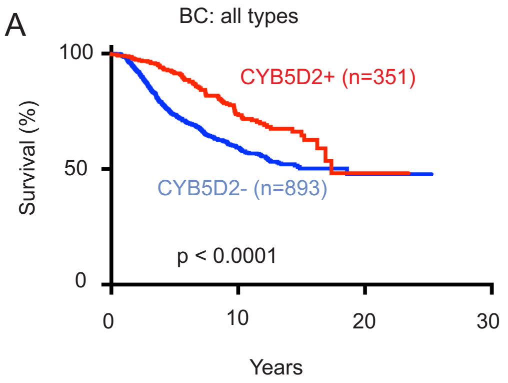

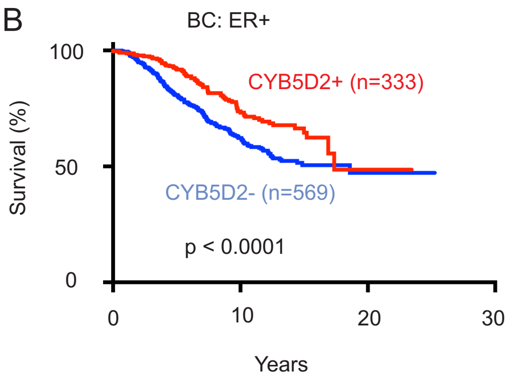

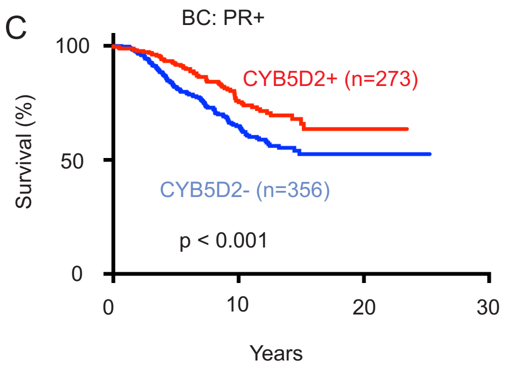

Figure S3. Downregulation of CYB5D2 correlates with reductions in overall survival (OS) in patients with breast cancer. The CYB5D2 mRNA expression data and survival information in a follow-up period approximately 30 years were extracted from the Curtis dataset. The effects of CYB5D2 downregulation on OS for BC and the indicated subtypes were evaluated using Kaplan-Meier curve. Statistical analysis was performed using Logrank Test.

Figure S4

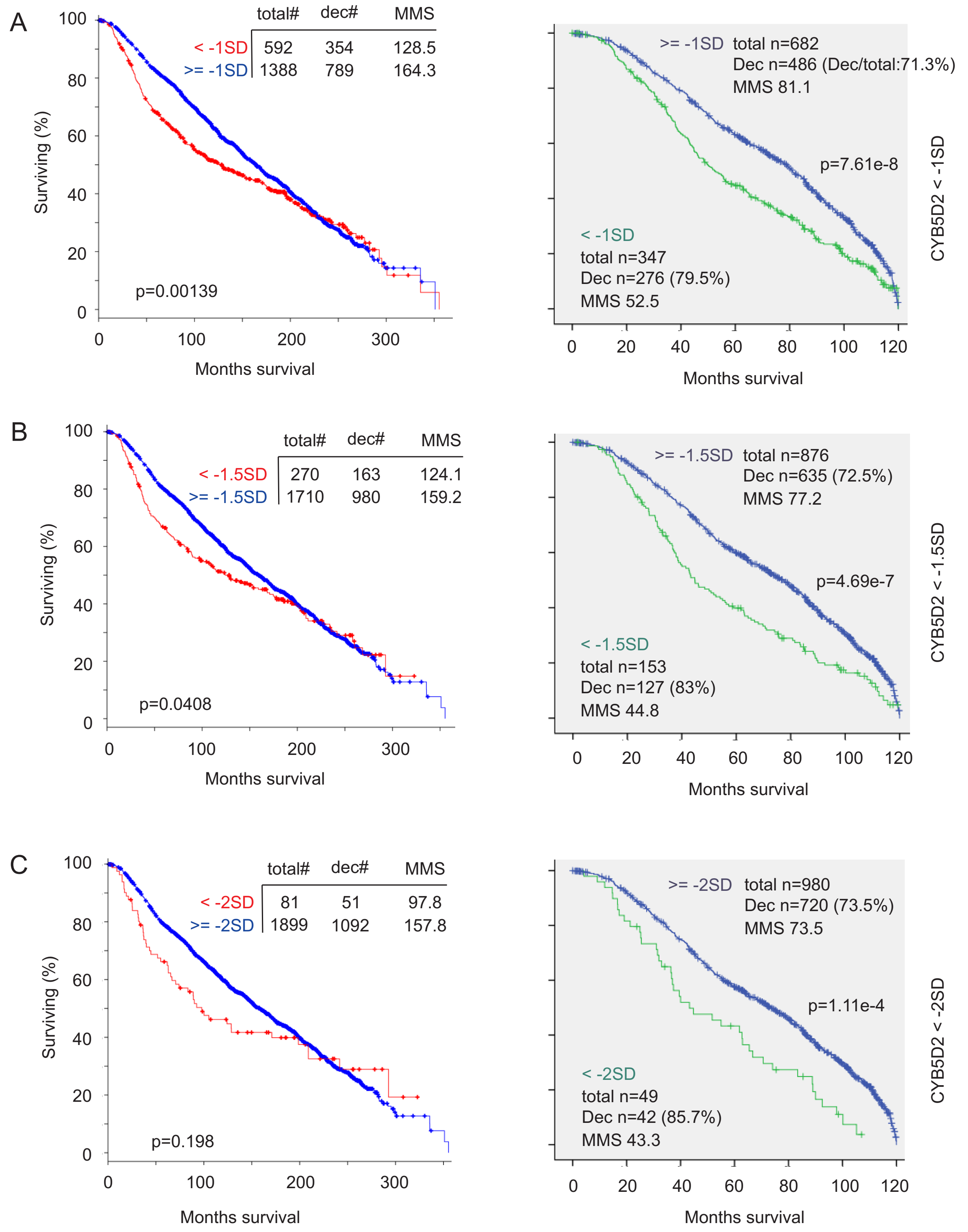

Figure S4. Characterization of CYB5D2 downregulation-associated shortening of OS. Data were extracted from the Curtis dataset [6] within the cBioPortal database. The impact of CYB5D2 mRNA reduction at level less than 1SD (A), 1.5SD (B) and 2SD (C) from the reference population mean on OS for patients with breast cancer (n=1980) was analyzed. Statistical analysis was performed using Logrank Test. Total#: total number of cases; dec#: number of deceased cases; MMS: median months survival. The follow-up period was for 350 months (left panels) and 120 months (right panels), respectively.

Figure S5

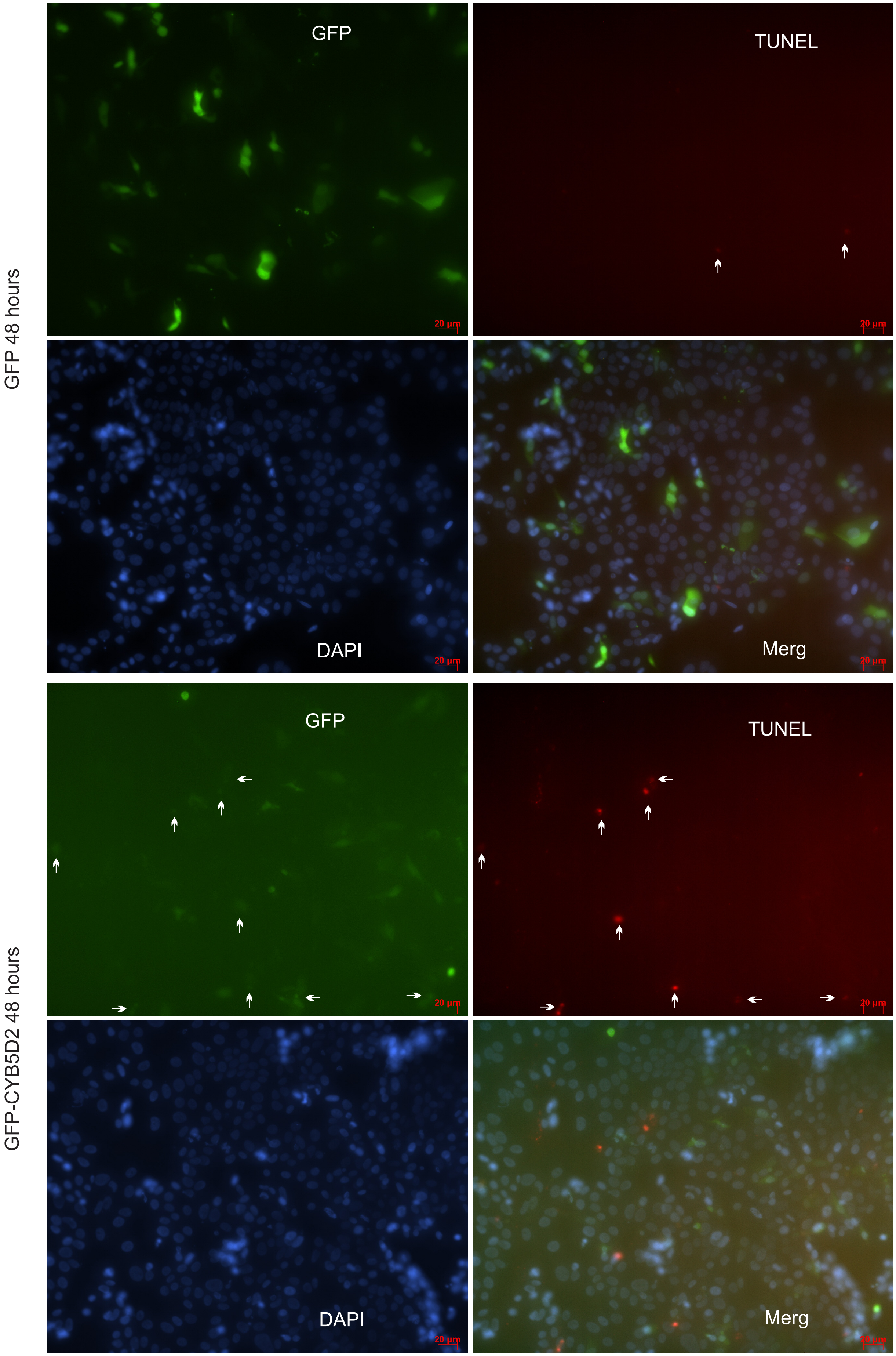

Figure S5. CYB5D2 expression induced apoptosis in MCF7 cells. MCF7 cells were transiently transfected with GFP or GFP-CYB5D2 for 48 hours, followed by TUNEL staining. Experiments were repeated three times; typical images are shown. Arrows indicated the corresponding GPF and TUNEL positive cells.

Figure S6

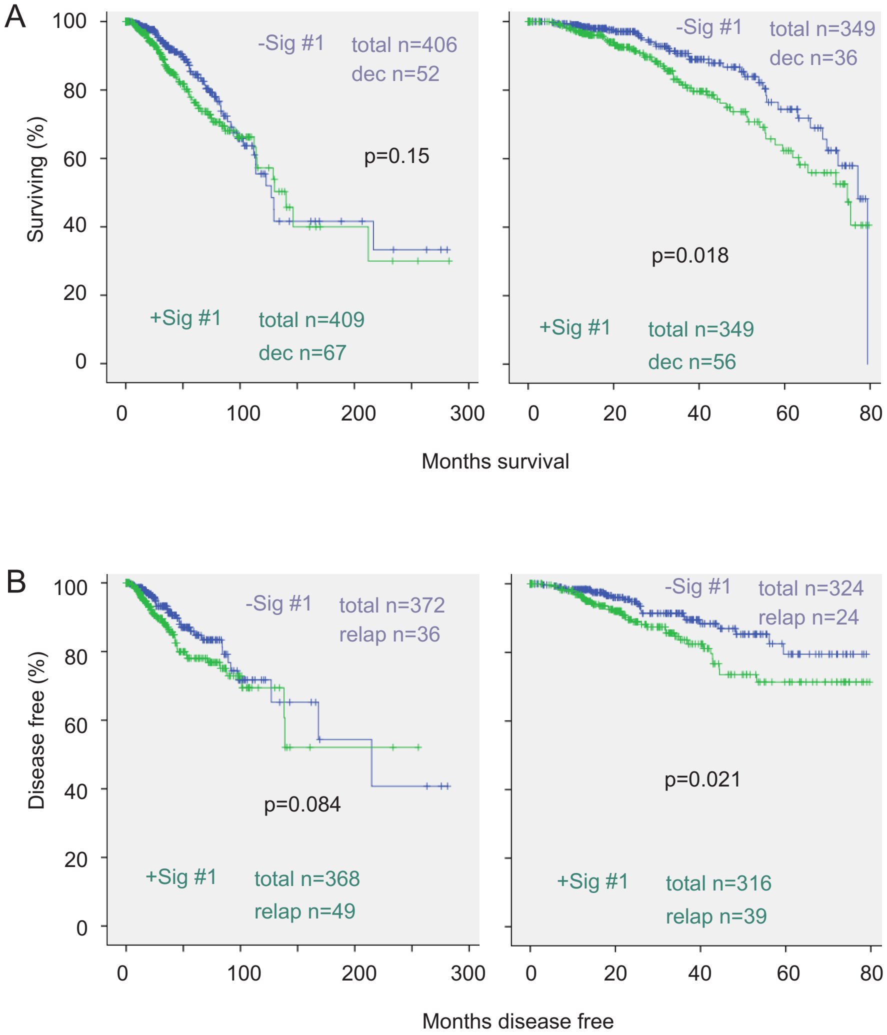

Figure S6. Signature #1 associates with decreases in OS and disease free survival (DFS) in patients with breast cancer. Data were extracted from the TCGA-Cell dataset (n=817). The effects of Signature #1 on OS (**A**) and DFS (**B**) in the indicated follow-up period of 300 and 80 months are determined. Statistical analysis was performed using Logrank Test. dec: deceased cases; relap: relapse cases.

Figure S7

Signature #2

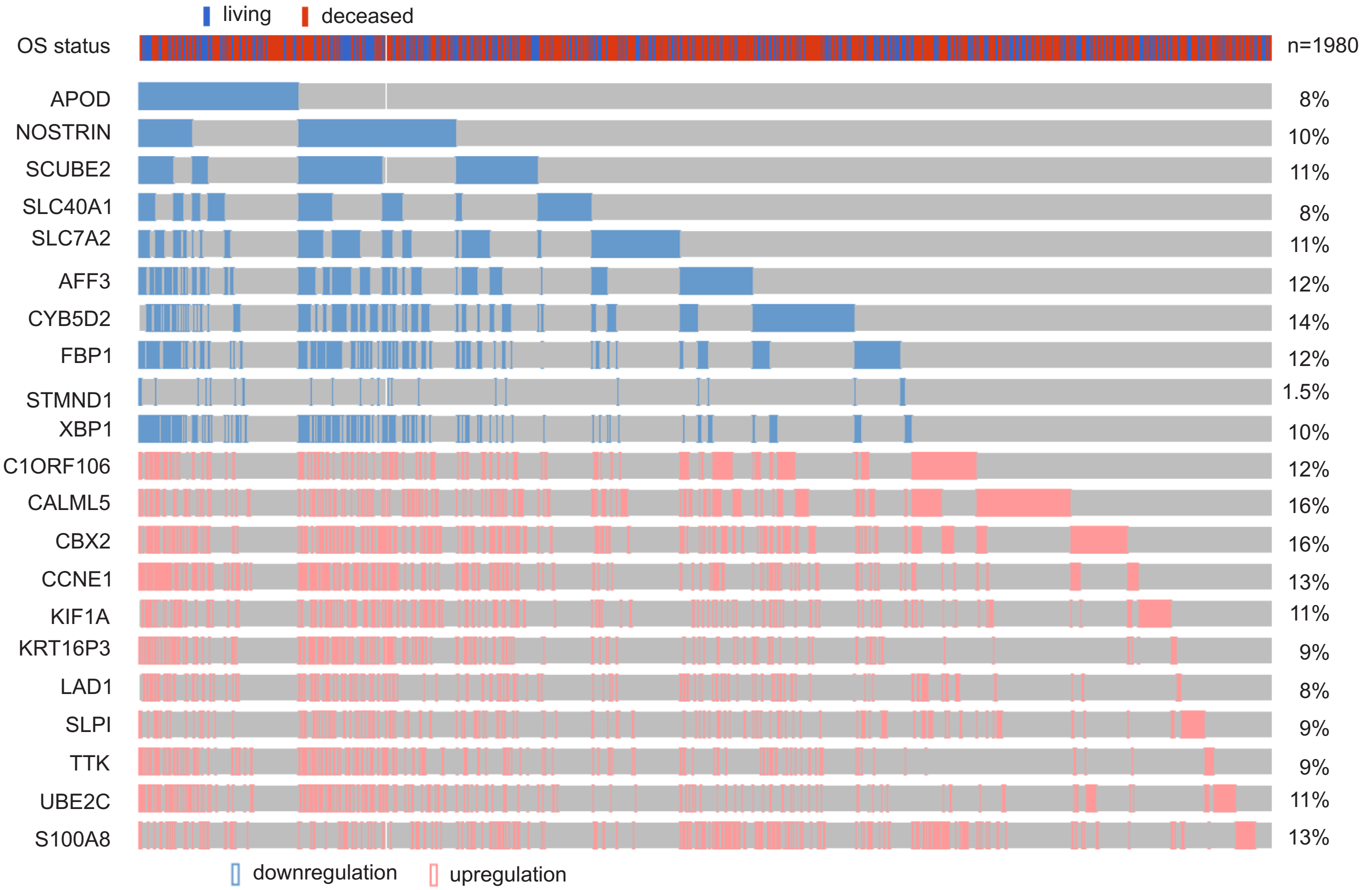

Figure S7. Alterations in Signature #2 in breast cancer. Data were derived from the Curtis dataset. Gene expression for the indicated genes along with OS status are included.

Figure S8

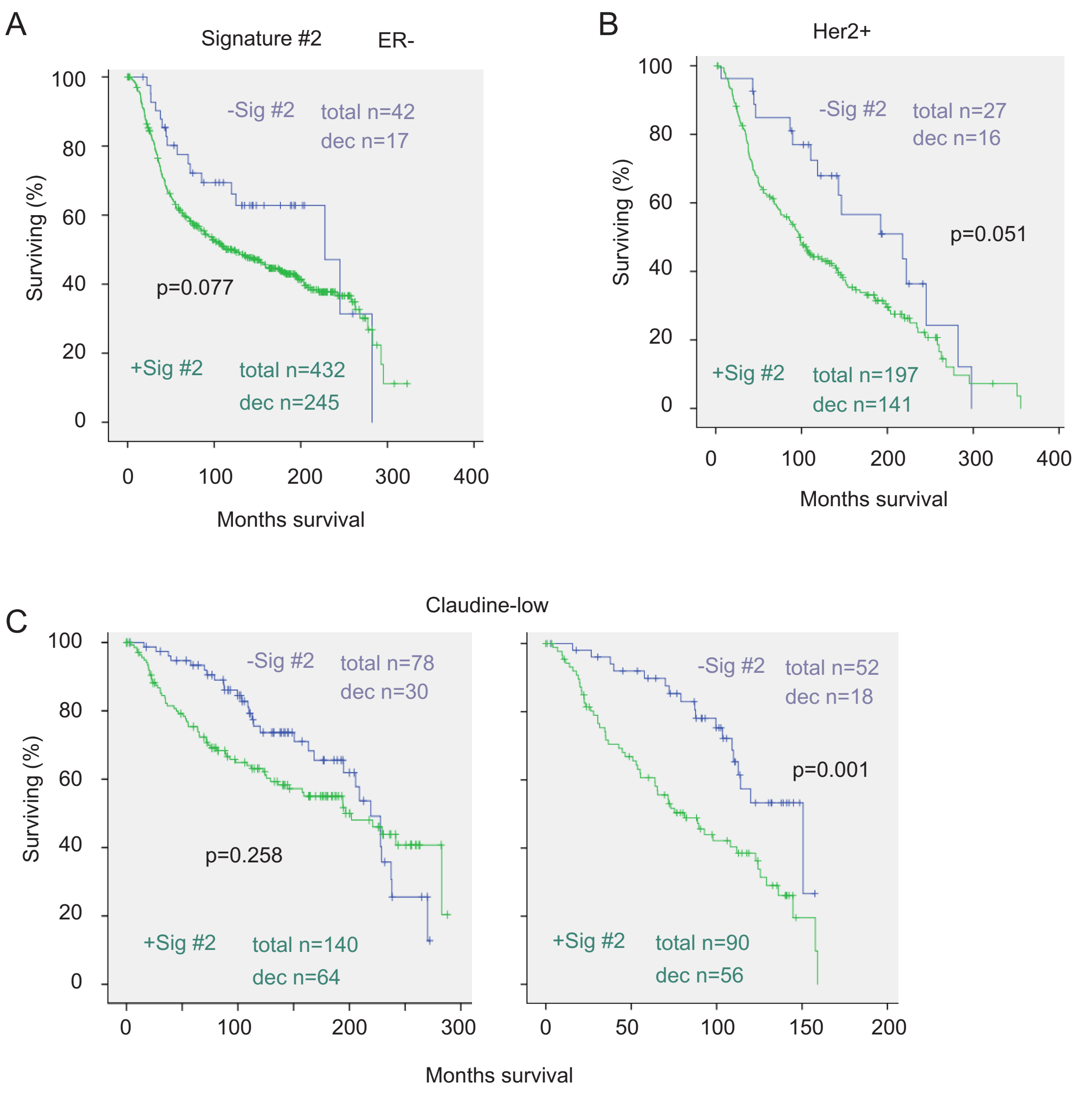

Figure S8. Characterization of Signature #2 with respect to OS. Data were derived from the Curtis dataset (n=1980). The effects of Signature #2 on OS for patients with ER-negative (**A**), HER2+ (**B**), and normal-like (**C**) breast cancer within the indicated follow-up period were determined. Statistical analysis was performed using Logrank Test. dec: deceased cases.

Figure S9

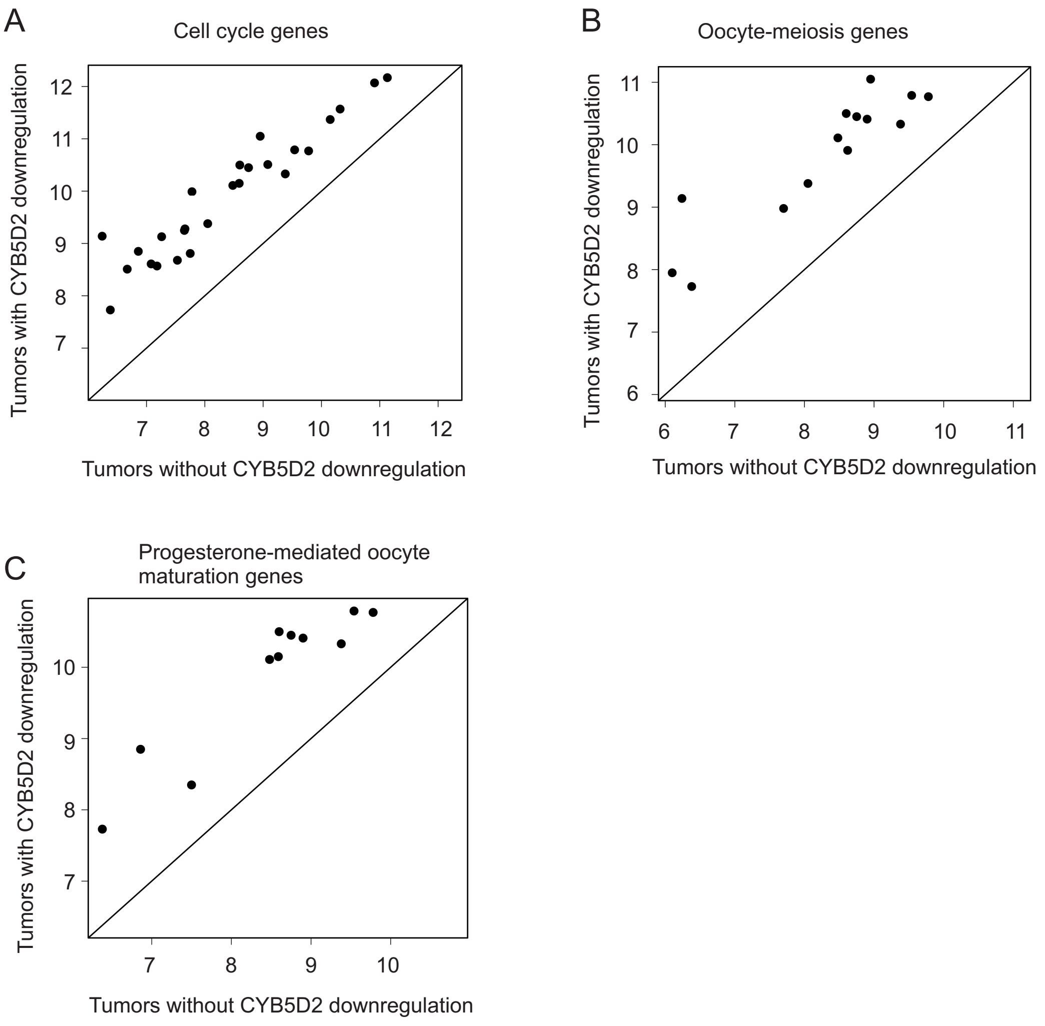

Figure S9. Elevations in gene expression in the enriched gene sets in common-DEGs. Enrichment analysis for gene sets with respect to common-DEGs was performed using the Gage package in R. Scatter plots show increases in expression for genes in a cell cycle gene set (A), oocyte-meiosis gene set (B), and progesterone-mediated oocyte maturation gene set. See Table S4A-C for the individual genes.

Figure S10

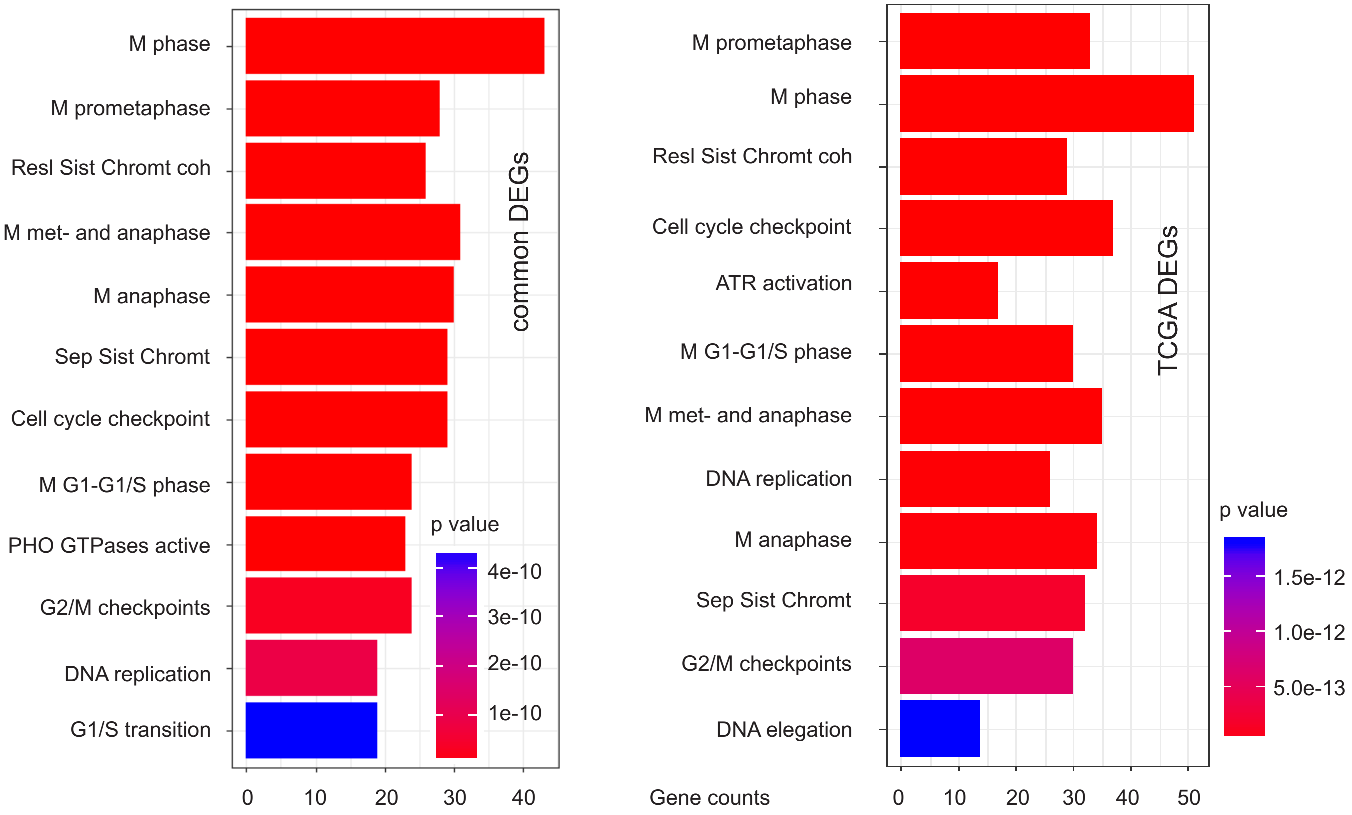

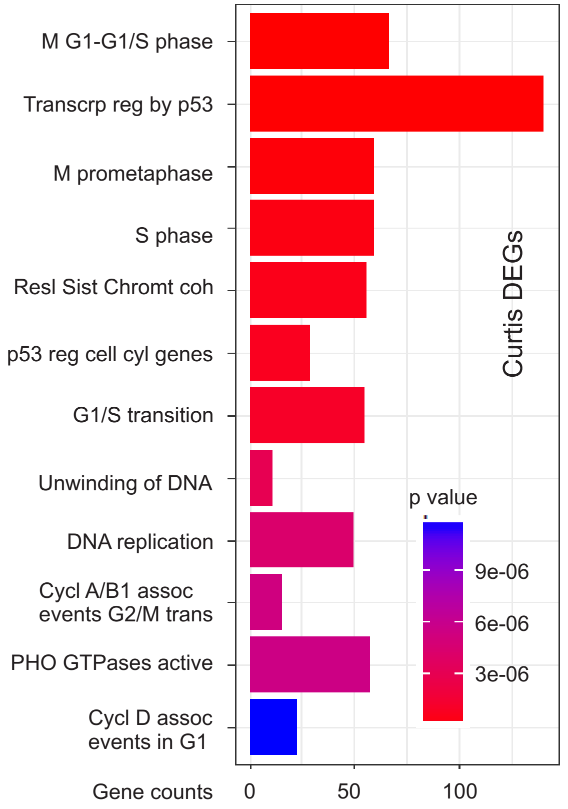

Figure S10. CYB5D2 downregulation-associated DEGs are enriched in pathways related to essential aspects of cell proliferation. Data on differentially expressed genes (DEGs) in CYB5D2 downregulated tumors were extracted from the TCGA-Cell (n=817) and Curtis (n=1980) datasets within the cBioPortal database. DEGs occurred in both datasets (common-DEGs) were identified (see Table S3C for details). Pathway enrichment for the three populations of DEGs was performed using the R Reactome package. Barplots for the top 12 enriched pathways are generated .

Figure S11

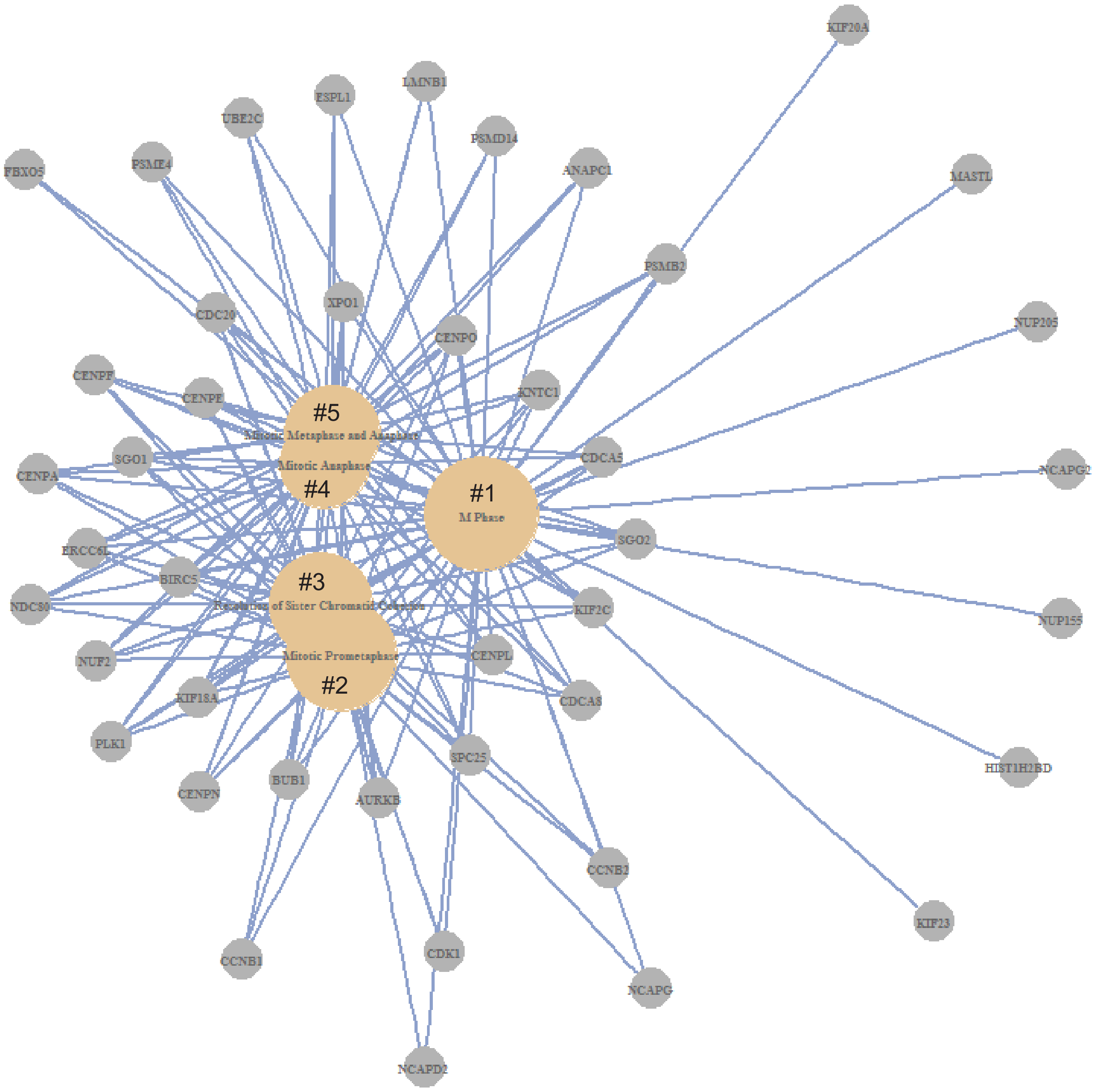

Figure 11. Relationship of specific common-DEGs with those of enriched pathways. Analysis for pathway enrichment was carried out using the Reactome package in R. Enriched individual pathways are placed in centers with the connected individual DEGs at outside. The enriched pathways are for M Phase (#1), Mitotic Prometaphase (#2), Resolution of Sister Chromatid Cohesion (#3), Mitotic Anaphase (#4), and Mitotic Metaphase and Anaphase (#5).

Figure S12

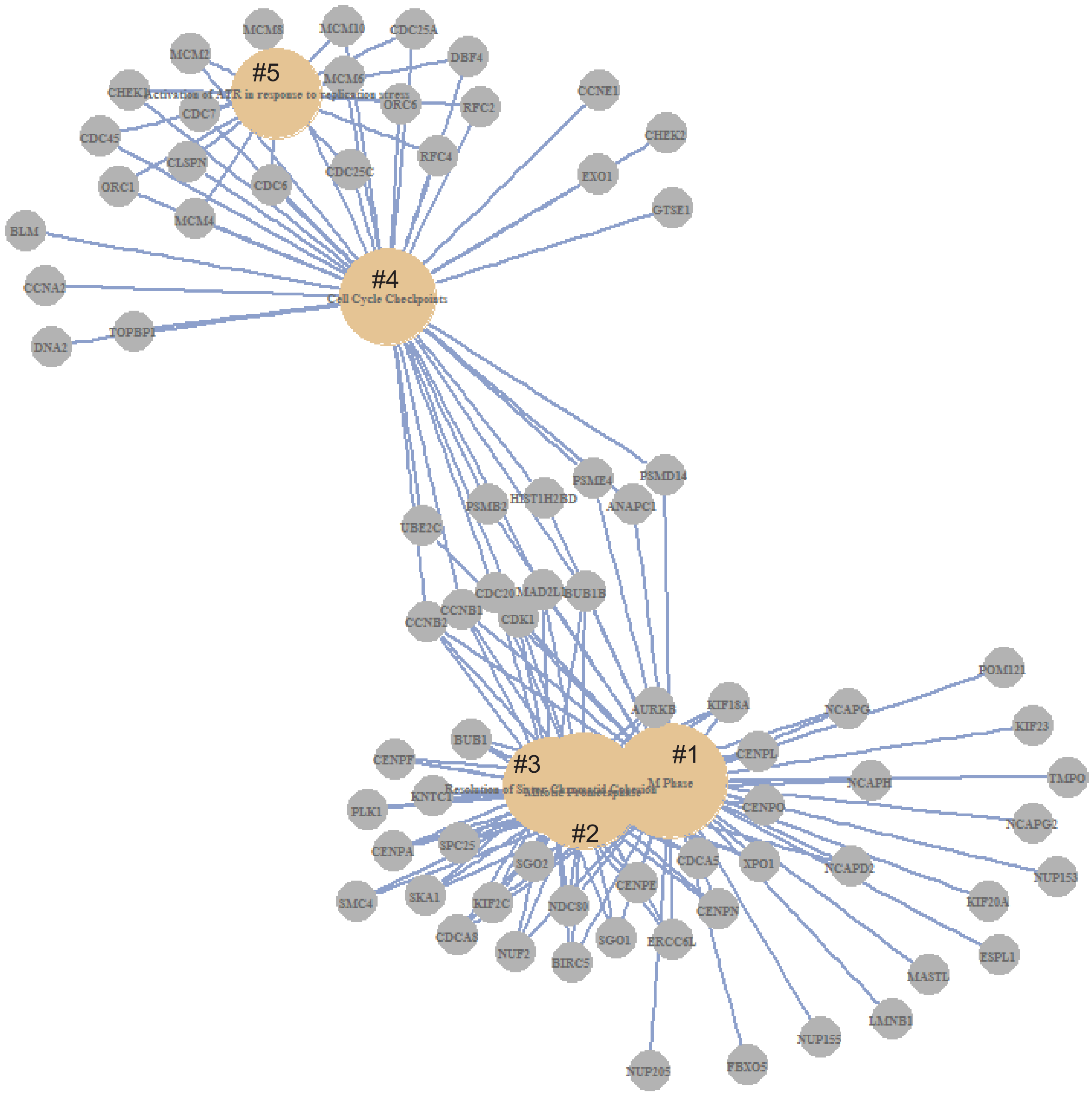

Figure S12. Relationship of specific TCGA-Cell-DEGs with those of enriched pathways. Analysis for pathway enrichment was performed using the Reactome package in R. Enriched individual pathways are placed in centers with the connected individual DEGs at outside. The enriched pathways are for M Phase (#1), Mitotic Prometaphase (#2), Resolution of Sister Chromatid Cohesion (#3), Cell Cycle Checkpoints (#4), and Checkpoint activation of ATR in response to replication stress (#5).

Figure S13

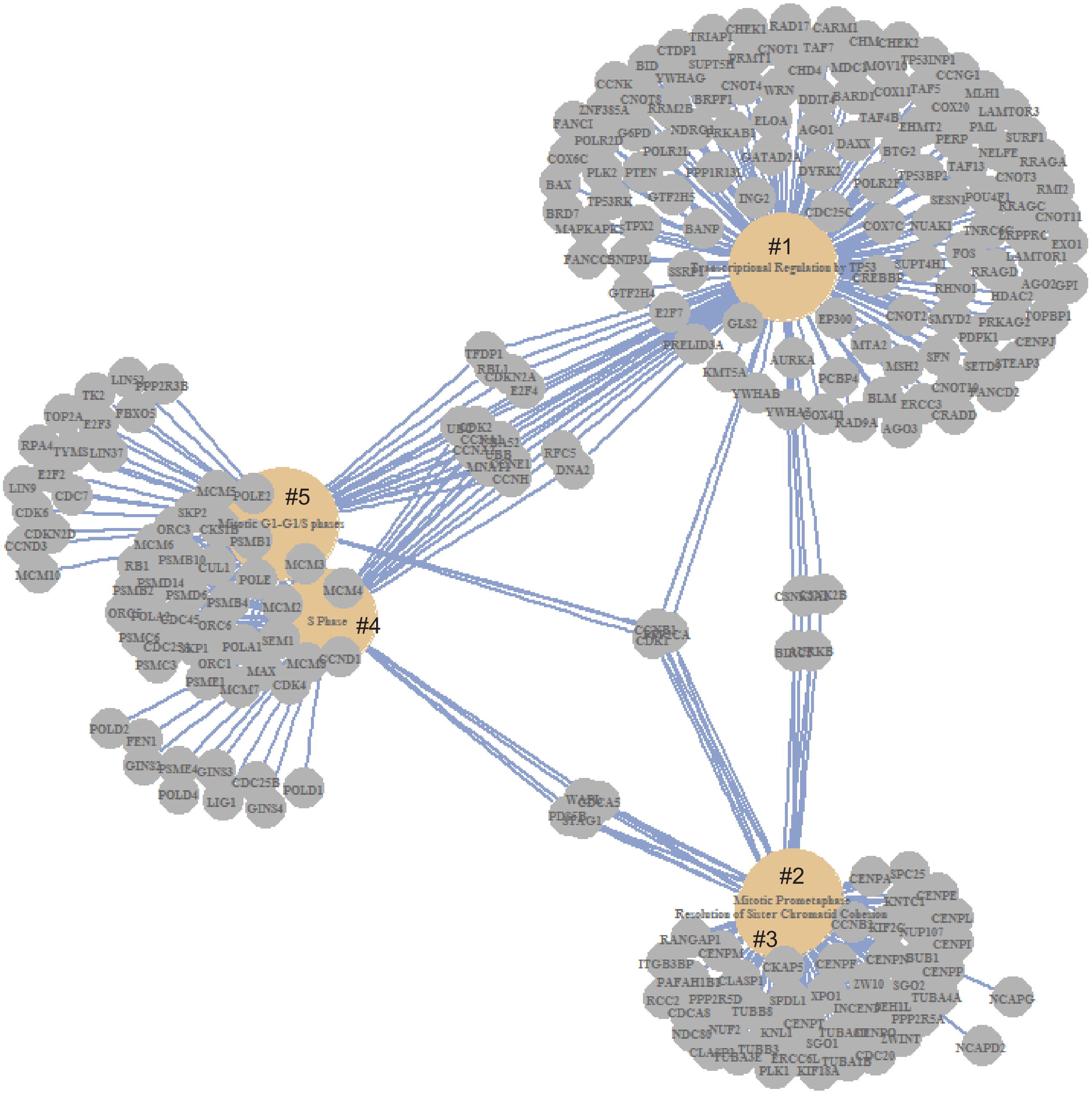

Figure S13. Relationship of specific Curtis-DEGs with those of enriched pathways. Analysis for pathway enrichment was performed using the Reactome package in R. Enriched individual pathways are placed in centers with the connected individual DEGs at outside. The enriched pathways are for Transcription Regulation by TP53 (#1), Mitotic Prometaphase (#2), Resolution of Sister Chromatid Cohesion (#3), S Phase (#4), and Mitotic/G1 – G1/S phase (#5).

Figure S14

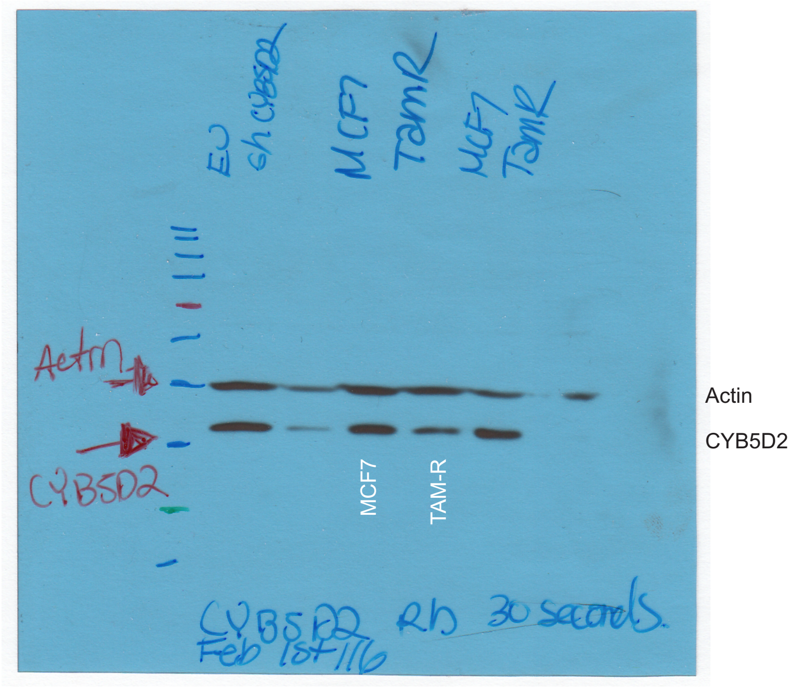

Figure S14. The intact Western blot from which the Fig 1B inset was derived. Note: the relevant lanes are the 3rd (MCF7 cells) and fourth (TamR cells) lanes from the left marker lane. The top and bottom bands are for Actin and CYB5D2, respectively.

Figure S15

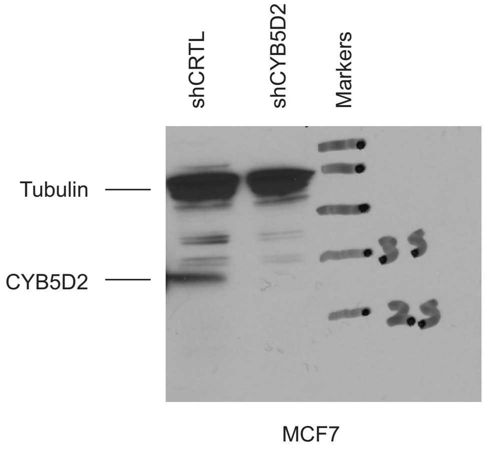

Figure S15. The intact Western blot from which the Fig 2D inset was derived.

Table S1. Co-alterations in mutations with CYB5D2 mRNA downregulations [a]

Gene	locus	Altered[b]	Unchanged[b]	Log R[c]	p-value	q-value
TP53	17q13.1	70 (86.4%)[d,f-h]<br>217 (80.6%)[g,h]<br>404 (68.8%)[h]	794 (34.7%)[e]<br>647 (30.8%)<br>460 (25.8%)	1.32<br>1.39<br>1.41	4.23e-21<br>2.95e-56<br>1.73e-77	7.32e-19<br>5.11e-54<br>1.34e-74
CDH1	16q22.1	0 (0%)[f-g]<br>5 (1.86%)[g,h]<br>37 (6.3%)[h]	236 (10.3%)<br>231 (11.0%)<br>199 (11.2%)	<-10<br>-2.57<br>-0.83	1.74e-4<br>3.61e-8<br>2.62e-4	0.015<br>2.08e-6<br>5.66e-3
GATA3	10p11	1 (1.23%) [f-g]<br>5 (1.86%) [g,h]<br>26 (4.4%) [h]	268 (11.7%)<br>264 (12.6%)<br>243 (13.6%)	-3.25<br>-2.76<br>-1.62	5.67e-4<br>8.42e-10<br>3.05e-11	0.033<br>7.29e-8<br>2.63e-9
PIK3CA	3q26.3	71 (26.4%) [g,h]<br>191 (32.5%) [h]	904 (43.1%)<br>786 (44%)	-0.71<br>-0.44	6.52e-8<br>1.58e-7	2.82e-6<br>2.21e-5
CBFB	16q22.1	0 (0%) [g,h]<br>13 (2.2%)	109 (5.19%)<br>96 (5.4%)	<-10<br>-1.28	1.42e-6<br>5.37e-4	4.90e-5<br>9.29e-3
MAP3K1	5q11.2	11 (4.1%) [g,h]<br>25 (4.3%) [h]	225 (10.7%)<br>211 (11.8%)	-1.39<br>-1.48	1.47e-4<br>7.93e-9	4.25e-3<br>4.59e-7
MUC16	19p13.2	135 (23%) [h]	274 (15.4%)	0.58	2.56e-5	7.81e-4
SYNE1	6q25	101 (17.2%) [h]	193 (10.8%)	0.67	5.24e-5	1.51e-3
AKAP9	7q21-22	56 (9.5%) [h]	82 (4.9%)	0.97	6.14e-5	1.52e-3
RB1	13q14.2	26 (4.3%) [h]	30 (1.7%)	1.4	2.99e-4	5.75e-3
CACNA2D3	3p21.1	18 (3.1%) [h]	18 (1%)	1.6	8.12e-4	0.0131
CTCF	16q21-22.3	4 (0.68%) [h]	50 (2.8%)	-2.04	9.8e-4	0.0131
BRCA1	17q21	20 (3.4%) [h]	22 (1.2%)	1.46	1.0e-4	0.0134
RYR2	1q43	57 (9.1%) [h]	104 (5.8%)	0.73	1.77e-3	0.0145
AHNAK2	14q32.33	120 (20.4%) [h]	275 (15.4%)	0.41	3.27e-3	0.0355
CASP8	2q33-34	14 (2.4%) [h]	14 (0.8%)	1.6	3.81e-3	0.0355
THSD7A	7p21.3	25 (4.3%) [h]	36 (2%)	1.08	3.5e-3	0.0356
BIRC6	2p22.3	45 (7.7%) [h]	83 (4.7%)	0.72	4.52e-3	0.434

a: CYB5D2 mRNA reduction at levels < -1SD, -1.5SD, and -2SD (standard derivation); co-alterations in mutation were derived from the Metabric dataset (n=2509) within the cBioPortal database

b: altered (with the indicated CYB5D2 downregulations) and unchanged (without the indicated CYB5D2 downregulations) group

c: log2-based ratio of percentage in altered group/percentage in unchanged group; positive and negative ratios are for co-occurrence and mutual exclusiveness, respectively

d: number of mutation cases/number of cases with CYB5D2 downregulation x100

e: number of mutation cases/number of cases without CYB5D2 downregulation x100

f-h: downregulation of CYB5D2 mRNA < -2SD (f), -1.5SD (g), and -1SD (h), respectively

p-values were determined by Fisher Exact Test (cBioPortal)

q-value were derived from Benjamini-Hochberg procedure (cBioPortal)

Table S2. Components of Signature #1 and their functions

Gene	locus	Function	Reference
CYB5D2	17p13.2	Potential tumor suppressor	Xie et al, 2016
TP53	17p13.1	Tumor suppressor	Well-established
CDH1	16q22.1	Tumor suppressor	Well-established
BRCA1	17q21	Tumor suppressor	Well-established
THSD7A<br>Thrombospondin Type-1 Domain containing 7A	7p21.3	-Promotes endothelial cell migration and filopodia formation during angiogenesis<br>-Potential tumor antigen	Wang et al, 2010<br>Kuo et al. 2011<br>Stahl et al, 2017
BIRC6<br>Baculoviral IAP repeat containing 6	2p22.3	Antiapoptotic protein	Low et al., 2013<br>Tang et al., 2015
RB1	13q14.2	Tumor suppressor	Well-established

Kuo, M.W., Wang, C.H., Wu, H.C., Chang, S.J. and Chuang, Y.J., 2011. Soluble THSD7A is an N-glycoprotein that promotes endothelial cell migration and tube formation in angiogenesis. *PloS one, 6*(12), p.e29000.

Low, C.G., Luk, I.S., Lin, D., Fazli, L., Yang, K., Xu, Y., Gleave, M., Gout, P.W. and Wang, Y., 2013. BIRC6 protein, an inhibitor of apoptosis: role in survival of human prostate cancer cells. *PLoS one, 8*(2), p.e55837.

Stahl, P.R., Hoxha, E., Wiech, T., Schröder, C., Simon, R. and Stahl, R.A., 2017. THSD7A expression in human cancer. *Genes, Chromosomes and Cancer*, *56*(4), pp.314-327.

Tang, W., Xue, R., Weng, S., Wu, J., Fang, Y., Wang, Y., Ji, L., Hu, T., Liu, T., Huang, X. and Chen, S., 2015. BIRC6 promotes hepatocellular carcinogenesis: Interaction of BIRC6 with p53 facilitating p53 degradation. *International journal of cancer*, *136*(6), pp.E475-E487.

Wang, C.H., Su, P.T., Du, X.Y., Kuo, M.W., Lin, C.Y., Yang, C.C., Chan, H.S., Chang, S.J., Kuo, C., Seo, K. and Leung, L.L., 2010. Thrombospondin type I domain containing 7A (THSD7A) mediates endothelial cell migration and tube formation. *Journal of cellular physiology*, *222*(3), pp.685-694.

Xie, Y., Shen, Y.T., Kapoor, A., Ojo, D., Wei, F., De Melo, J., Lin, X., Wong, N., Yan, J., Tao, L. and Major, P., 2016. CYB5D2 displays tumor suppression activities towards cervical cancer. *Biochimica et Biophysica Acta (BBA)-Molecular Basis of Disease*, *1862*(4), pp.556-565.
.
